# AAV9/*SLC6A1* gene therapy rescues abnormal EEG patterns and cognitive behavioral deficiencies in *Slc6a1*^–/–^ mice

**DOI:** 10.1172/JCI182235

**Published:** 2024-11-26

**Authors:** Weirui Guo, Matthew Rioux, Frances Shaffo, Yuhui Hu, Ze Yu, Chao Xing, Steven J. Gray

**Affiliations:** 1 Department of Pediatrics,; 2 McDermott Center for Human Growth and Development,; 3 Department of Bioinformatics,; 4 O’Donnell School of Public Health,; 5 Department of Neurology,; 6 Department of Molecular Biology, and; 7 O’Donnell Brain Institute, University of Texas Southwestern Medical Center, Dallas, Texas, USA.

**Keywords:** Therapeutics, Neurological disorders

## Abstract

The solute carrier family 6 member 1 (*SLC6A1*) gene encodes the γ-aminobutyric acid (GABA) transporter GAT-1, the deficiency of which is associated with infantile encephalopathy with intellectual disability. We designed 2 AAV9 vectors, with either the JeT or MeP promoter, and conducted preclinical gene therapy studies using heterozygous and homozygous *Slc6a1*-KO mice at different developmental ages and various routes of administration. Neonatal intracerebroventricular administration of either vector resulted in significantly normalized EEG patterns in *Slc6a1^–/–^* or *Slc6a1^+/–^* mice as well as improvement in several behavioral phenotypes of *Slc6a1^–/–^* mice. However, some mortality and adverse effects were observed in neonatal-treated mice. Intrathecal administration of either vector at P5 normalized EEG patterns in *Slc6a1^+/–^* mice, but in *Slc6a1^–/–^* mice, the treatment only rescued nest building without impact on EEG. Both vectors were well tolerated in all mice treated at P5 or later (including WT mice), up to 1 year after injection. Overall, our data demonstrate compelling efficacy when mice are treated at an early development age. We also identified that outside of the neonatal treatment window, the severe homozygous KO model is more refractory to treatment, whereas our treatments in the heterozygous mice, which genotypically match human patients, have resulted in stronger benefits.

## Abstract 2

AAV9/*SLC6A1* gene therapy rescues abnormal EEG patterns and cognitive behavioral deficiencies in *Slc6a1*-/- mice

## Graphical abstract

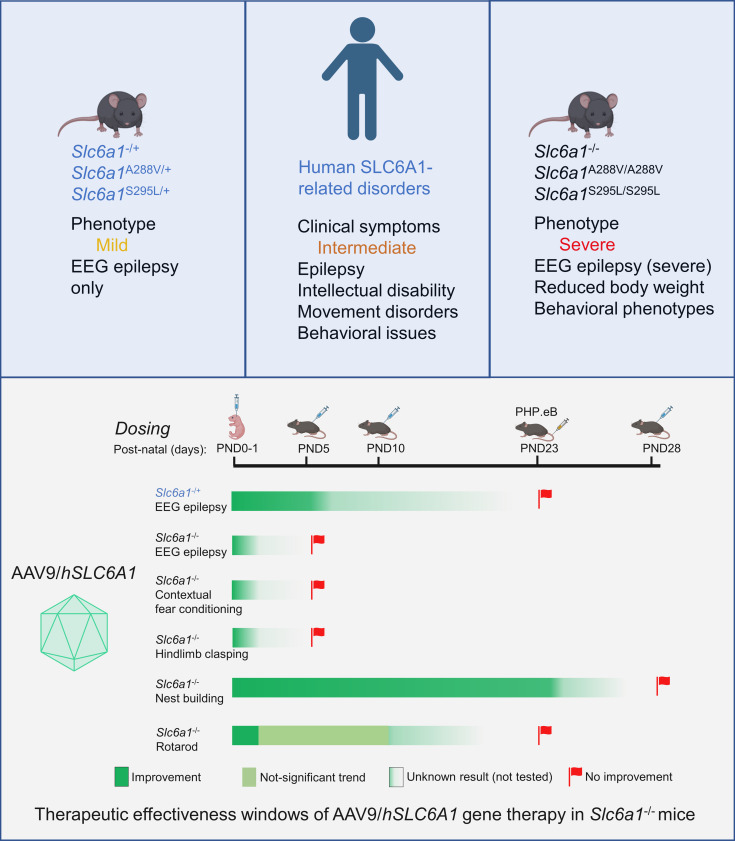

## Introduction

The solute carrier family 6 member 1 (*SLC6A1*) related disorders constitute a group of rare neurodevelopmental syndromes with early childhood onset of phenotypes, featuring epilepsy, intellectual disability, movement disorders, and behavior resembling autism spectrum disorder (ASD) (1). Initially identified in 2015 within a group of patients with Doose syndrome, the range of phenotypes linked to *SLC6A1*-related disorders has since broadened. The estimated prevalence of these disorders is approximately 1 in 38,000 births (2–4).

*SLC6A1*-related disorders typically result from a pathogenic genetic variant in 1 copy of the *SLC6A1* gene, which encodes the voltage-dependent γ-aminobutyric acid (GABA) transporter type 1 (GAT-1). GAT-1 is responsible for GABA reuptake from presynaptic nerve terminals as well as glial cells (5–7). At present, there are more than 200 known pathogenic variants in *SLC6A1*, with the majority being de novo mutations (8, 9). The mechanisms through which these pathogenic variants lead to clinical manifestations are not well understood. The anticipated molecular mechanism involves the loss of function resulting in haploinsufficiency (10, 11).

Similar to other neurodevelopmental disorders, *SLC6A1*-related disorders currently lack effective treatments. The available interventions primarily focus on symptom control, involving the administration of antiseizure medications, supportive developmental therapies, and medications aimed at mitigating the impact of symptoms (12). Notably, there is no observed correlation between seizure control and cognitive behavioral outcomes (1).

Over the past 20 years, there has been substantial exploration of viral vector-based gene therapy as a potential treatment for genetic disorders. In numerous studies, the recombinant adeno-associated viral vector type 9 (AAV9) has consistently demonstrated both safety and efficacy as a vehicle for delivering transgenic cargo to the central nervous system (CNS) (13, 14). AAV9 has become the preferred choice for CNS gene delivery due to its capacity to facilitate broad transgene expression across the CNS tissues of rodents and larger animal models following injection in the cerebrospinal fluid (CSF) (15). Moreover, intrathecal AAV9 gene therapy is currently being employed in active clinical trials for conditions such as giant axonal neuropathy, CLN7 Batten disease, Rett syndrome, and spastic paraplegia 50 (SPG50) disease, among others (16–20).

In this research, we assessed and selected the most suitable mouse model from *Slc6a1*-KO and patient variant knockin (KI) mice to recapitulate the disease symptoms of *SLC6A1*-related disorders observed in humans. We then designed and tested AAV9 vectors carrying the human *SLC6A1* (*hSLC6A1*) transgene in the KO mouse model to evaluate the efficacy and safety of AAV9/*hSLC6A1* gene transfer across different development ages.

## Results

### The constitutive Slc6a1-KO mouse model and 2 KI mouse models of human patient variants S295L and A288V similarly recapitulate many features of human SLC6A1-related disorders.

The *SLC6A1* point missense mutations S295L and A288V have been discovered in human *SLC6A1* patients with epilepsy, intellectual disability, and neurodevelopmental disorders (2, 21). Both missense mutations were previously characterized in vitro at the molecular level. The S295L variant displayed a complete loss of GABA uptake function and endoplasmic reticulum–associated degradation, while the A288V variant displayed a partial loss of GABA reuptake function in cultured cells (11). To consider the suitability of the S295L and A288V missense variants as animal models of *SLC6A1*-related disorders for gene-replacement therapy, we conducted detailed phenotyping of 2 transgenic mouse lines carrying each variant (22, 23). We compared the body weight (BW), behavior, and EEG profile of each variant to the previously characterized B6.129S1 KO mouse (24–26).

**Figure 1:**
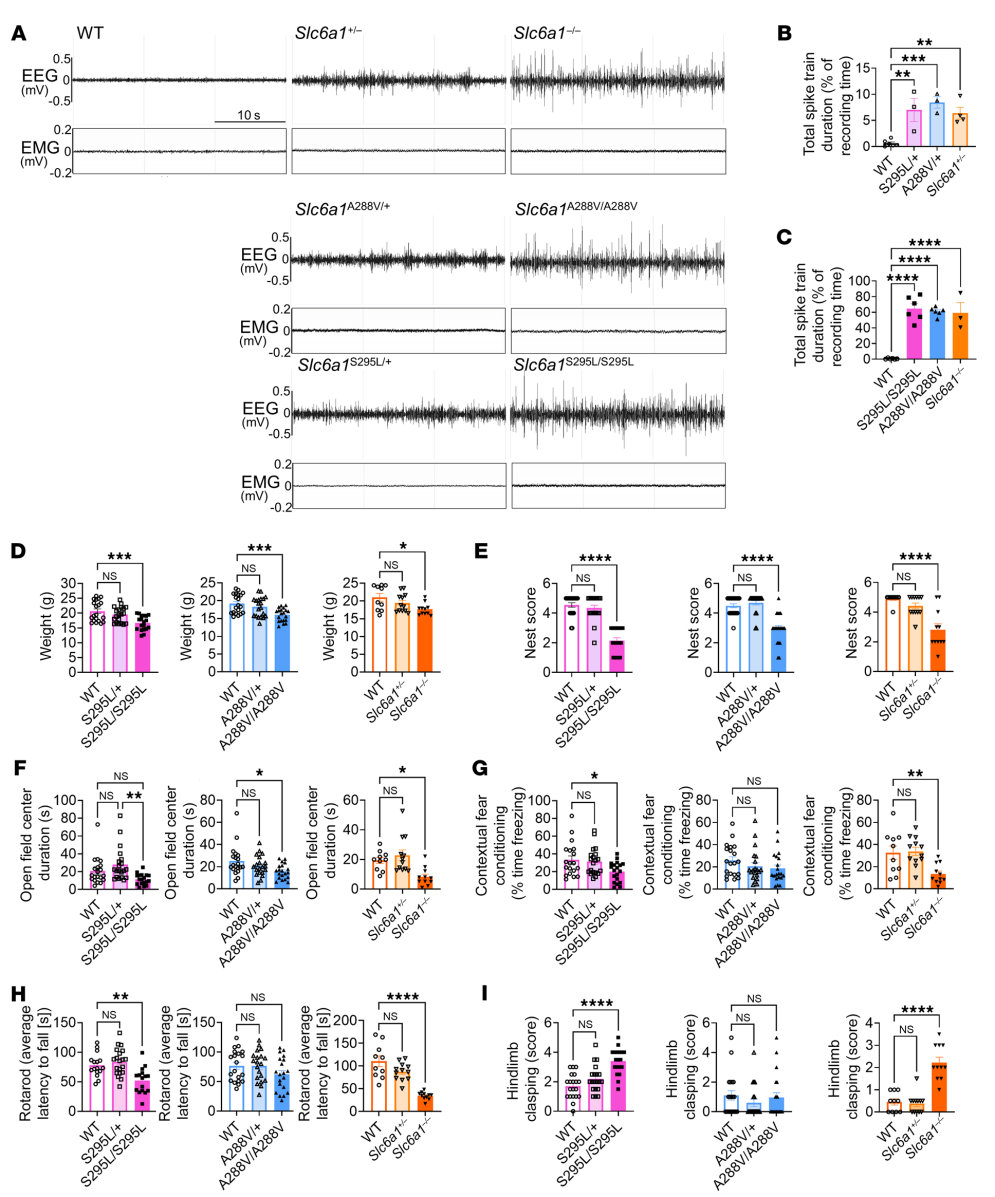
The constitutive *Slc6a1* KO mouse model and 2 KI mouse models of human patient variants S295L and A288V similarly phenocopy human SLC6A1-related disorders. All assessments were conducted in WT, heterozygous (*Slc6a1*^+/–^, *Slc6a1*^S295L/+^, *Slc6a1*^A288V/+^), and homozygous (*Slc6a1*^–/–^, *Slc6a1*^S295L/S295L^, *Slc6a1*^A288V/A288V^) *Slc6a1* KO and KI mice. (**A**) Representative EEG and EMG recording examples. A 10 Hz high pass filter was used to smooth the EEG signal. (**B**) Quantification of total spike train duration across a 48-hour EEG recording period (% recording time) in WT and heterozygous *Slc6a1* KO and KI mice (*n* = 6, 3, 3, and 4, left to right on the panel). (**C**) Quantification of total spike train duration across a 48-hour EEG recording period (% recording time) in WT and homozygous *Slc6a1* KO and KI mice (*n* = 6, 6, 6, and 3, left to right on the panel). (**D**) BW quantification at 8 weeks old. (**E**) Nest-building scores. (**F**) Time spent in the center (s) of an open field. (**G**) Freezing percentage time during the contextual fear conditioning tests. (**H**) Average latency to fall (s, average of 8 trials) of the rotarod test. (**I**) Hindlimb clasping scores. (Sample number in **D**, **E**, and **G**–**I**: WT, *Slc6a1*^S295L/+^, *Slc6a1*^S295L/S295L^: *n* = 19–20 per group; WT, *Slc6a1*^A288V/+^, *Slc6a1*^A288V/A288V^: *n* = 20 per group; WT, *Slc6a1*^–/+^, *Slc6a1*^–/–^: *n* = 10–12 per group). Data are represented as means ± SEM. One-way ANOVA with Dunnett’s multiple-comparisons test was used for statistical analysis. *P < 0.05; **P < 0.01; ***P < 0.001; ****P < 0.0001.

We measured the phenotypes of heterozygous and homozygous S295L and A288V KI mouse models, in parallel with the constitutive heterozygous *Slc6a1*^+/–^ and homozygous *Slc6a1*^–/–^ KO (null) mouse models that lacked 1 or 2 copies of the Slc6a1 gene. Since abnormal EEG readings, including epileptiform discharges and seizures, are a hallmark of *SLC6A1*-related disorders, we used 48-hour EEG telemetry recordings of the mouse brain to monitor epileptiform activities and compare the 3 allelic models. The heterozygous *Slc6a1*^S295L/+^, *Slc6a1*^A288V/+^, and *Slc6a1*^+/–^ mice all showed a similar epileptic seizure phenotype with abundant 3–7 Hz and low amplitude polyspike trains (as shown in Figure 1, A and B, and Supplemental Figure 1; supplemental material available online with this article; https://doi.org/10.1172/JCI182235DS1). The homozygous *Slc6a1*^S295L/S295L^, *Slc6a1*^A288V/A288V^, and *Slc6a1*^–/–^ mice all showed a similarly stronger epileptic seizure phenotype, with abundant 3–7 Hz polyspike trains, higher spike amplitude, and much longer duration compared with the heterozygous mice (Figure 1, A and B, and Supplemental Figure 1). These polyspike trains are rarely observed in WT mice, though some exhibited low-amplitude spike trains with a very short duration (Figure 1, A–C, and Supplemental Figure 1). In *Slc6a1*^–/–^ mice, polyspike trains are often accompanied by a cessation of muscle activity, which is consistent with typical absence seizures (Supplemental Video 1). We further investigated the motor and cognitive phenotypes across the 3 mouse models (Figure 1 and Supplemental Figure 2). Similar to the *Slc6a1*^+/–^ mice, *Slc6a1*^S295L/+^ and *Slc6a1*^A288V/+^ mice did not show any statistically significant abnormal phenotypes compared with WT controls. However, in some tests, such as nest building and rotarod, the heterozygous mice had scores that trended lower than WT, suggesting possible mild phenotypes that could emerge with larger sample sizes. Mice homozygous for the S295L or A288V alleles phenocopied each other and the *Slc6a1*^–/–^ KO mice in BW, nest building, fear conditioning, and open-field tests (Figure 1, D–F). There were some differences in the models with respect to the distance traveled in open-field testing, rotarod, and hindlimb clasping. Compared with the *Slc6a1*^–/–^ KO mice, the *Slc6a1*^S295L/S295L^ KI mice had slightly better rotarod performance and lacked a significant reduction in distance traveled in the open-field test (Figure 1H and Supplemental Figure 2B). Interestingly, the partial loss of GABA uptake function (~25% of WT function, in vitro measured in cultured cells; ref. 11) in *Slc6a1*^A288V/A288V^ mice did not cause significant impairment in rotarod testing or induce a hind limb clasping phenotype (Figure 1, G–I, and Supplemental Figure 2C). Taken together, all 3 homozygous mouse models (*Slc6a1*^–/–^, *Slc6a1*^S295L/S295L^, and *Slc6a1*^A288V/A288V^) develop key behavior phenotypes that are consistent with the human *SLC6A1* disorders. Additionally, *Slc6a1*^–/–^, *Slc6a1*^S295L/S295L^, and *Slc6a1*^A288V/A288V^ displayed nearly identical EEG phenotypes. Due to the subtle difference of behavioral phenotypes in the homozygous mice, we have ranked the models, in order of severity (most to least) as *Slc6a1* KO, S295L KI, and A288V KI (Supplemental Table 1). Overall, the heterozygous *Slc6a1*^+/–^ KO and homozygous *Slc6a1*^–/–^ KO mice showed the most consistent and robust phenotypes and were thus chosen as the primary models for our gene replacement studies.

### AAV9/hSLC6A1 gene therapy via neonatal intracerebroventricular injection rescues brain epilepsy and key behavioral phenotypes in Slc6a1 heterozygous and homozygous KO mice.

**Figure 2:**
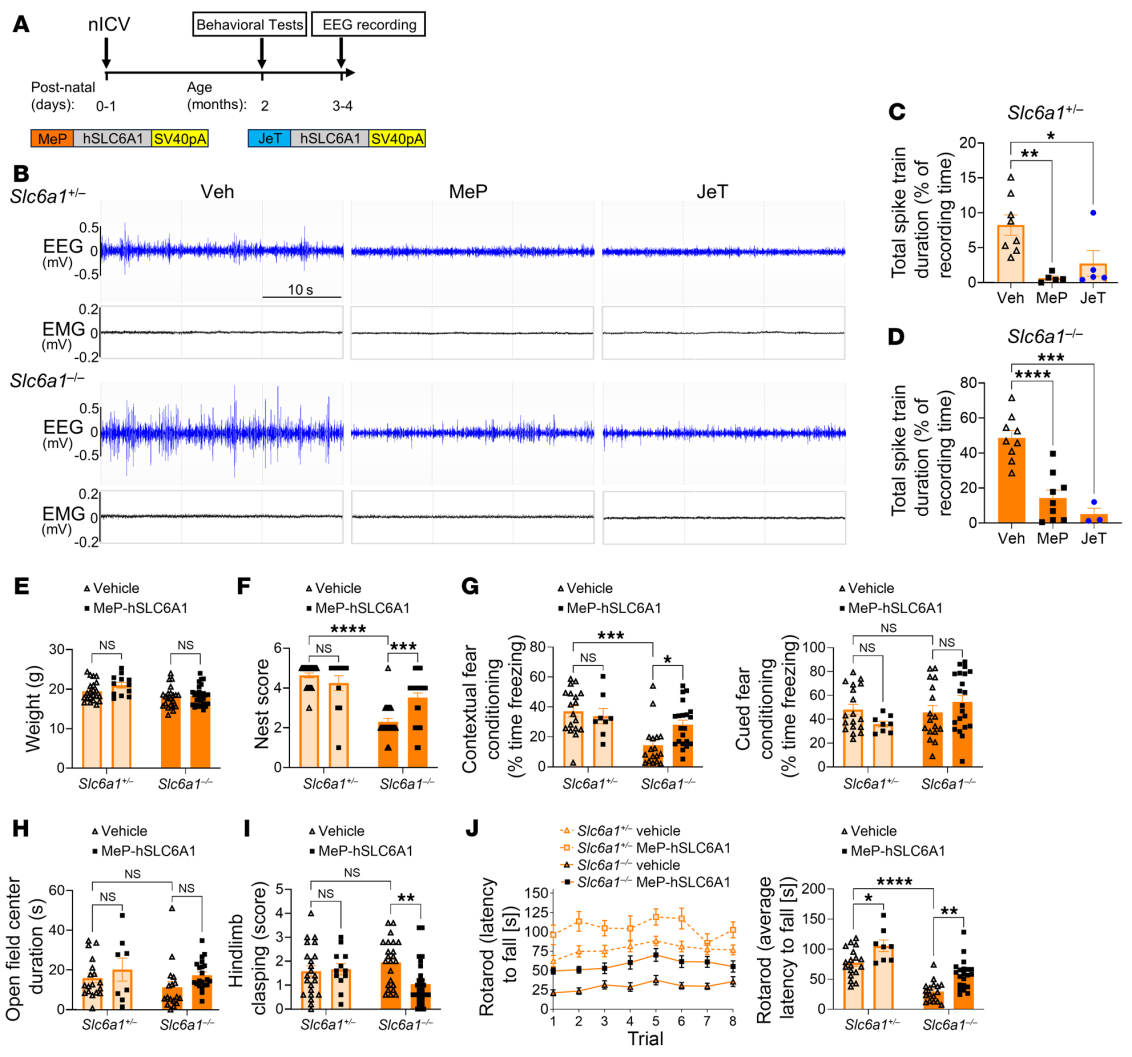
AAV9/*hSLC6A1* gene therapy rescues abnormal EEG patterns and key behavioral phenotypes in *Slc6a1* heterozygous and homozygous KO mice by neonatal ICV injection. (**A**) Experimental design of AAV9/*hSLC6A1* gene therapy via neonatal ICV injection (study plan, duration, and readouts). Three × 10^11^ vg AAV9/*hSLC6A1* vector or vehicle was administered to each mouse. Study readouts at each time point after dose administration or at the specified age are listed. Schematic diagram of the AAV9/*hSLC6A1* gene transfer cassettes using a MeP229 promoter (mostly neuron-specific promoter) or JeT promoter (weak ubiquitous promoter), the full-length codon-optimized human *SLC6A1* cDNA, and the SV40 poly(A) tail. (**B**) Representative EEG and EMG recording examples for heterozygous (*Slc6a1*^+/–^) and homozygous (*Slc6a1*^–/–^) mice injected with vehicle, AAV9/JeT-*hSLC6A1*, or AAV9/MeP229-*hSLC6A1*. A 10 Hz high pass filter was used to smooth the EEG signal. (**C**) Quantification of total spike train duration across a 48-hour EEG recording period (% recording time) of heterozygous mice injected with vehicle, AAV9/JeT-*hSLC6A1*, or AAV9/MeP229-*hSLC6A1* (*n* = 8, 5, and 5, left to right on the panel). (**D**) Quantification of total spike train duration across a 48-hour EEG recording period (% recording time) of homozygous mice injected with vehicle, AAV9/JeT-*hSLC6A1*, or AAV9/MeP229-*hSLC6A1* (*n* = 9, 9, and 3, left to right on the panel). Additional assessments in live *Slc6a1*^+/–^ and *Slc6a1*^–/–^ mice only compared vehicle and AAV9/MeP229-*hSLC6A1* treatment, after discontinuation of AAV9/JeT-*hSLC6A1* due to high mortality (panels **E**–**J**). (**E**) BWs (*n* = 22, 12, 22, 27; left to right on the panel), (**F**) nest-building scores (*n* = 22, 12, 22, 27; left to right on the panel), (**G**) contextual and cued fear conditioning freezing levels (*n* = 18, 8, 17, 21; left to right on the panel), (**H**) center time in an open field (*n* = 18, 8, 17, 21; left to right on the panel), (**I**) hind limb clasping scores (*n* = 22, 12, 22, 27; left to right on the panel), (**J**) rotarod latency to fall time (*n* = 18, 8, 17, 21; left to right on the panel). Data are represented as means ± SEM. One-way ANOVA with Dunnett’s multiple comparisons test was used for statistical analysis in **C** and **D**. Two-way ANOVA with Šidák’s multiple-comparisons test was used for statistical analysis in **E**–**J**. *P < 0.05; **P < 0.01; ***P < 0.001; ****P < 0.0001.

To test our hypothesis that broad CNS delivery of the human *SLC6A1* gene (*hSLC6A1*) using AAV9 can provide an effective treatment and long-term therapeutic benefit for SLC6A1-related disorders, we designed 2 AAV9 vectors carrying *hSLC6A1* driven by 2 different promoters and conducted preclinical gene-therapy studies using heterozygous (*Slc6a1*^+/–^) and homozygous (*Slc6a1*^–/–^) KO mice. One promoter is a weak universal promoter (JeT) (19, 27), whereas the other is a predominantly neuronal promoter (MeP229) (28) (Figure 2A). The 2 AAV9/*hSLC6A1* vectors were tested separately via bilateral intracerebroventricular (ICV) injection into neonatal P1 *Slc6a1*^+/–^ and *Slc6a1*^–/–^ mice (Figure 2A). We first investigated whether AAV9/*hSLC6A1* gene therapy could rescue the epileptic seizure phenotype previously characterized in *Slc6a1*^+/–^ and *Slc6a1*^–/–^ KO mice, which is a common feature of human *SLC6A1*-related disorders. Neonatal ICV administration of both JeT and MeP vectors (3 × 10^11^ vg per mouse) resulted in markedly reduced seizures, assessed by EEG in both *Slc6a1*^+/–^ and *Slc6a1*^–/–^ mice. In *Slc6a1*^+/–^ mice, both AAV9/MeP-*hSLC6A1* and AAV9/JeT-*hSLC6A1* reduced the duration of EEG spike trains to the extent that they were not significantly different from normal WT levels (Figure 2, B and C). In *Slc6a1*^–/–^ mice, the AAV9/MeP-*hSLC6A1* vector showed partial reduction of the EEG spike train duration to approximately 17%. The AAV9/JeT-*hSLC6A1* vector showed a greater degree of rescue in 3 treated *Slc6a1*^–/–^ KO mice (Figure 2, B and D). The AAV9/MeP-hSLC6A1 vector treatment in neonatal mice also ameliorated the fear conditioning, nest building, rotarod performance, and hind limb clasping phenotypes in *Slc6a1*^–/–^ KO mice. However, this treatment did not significantly improve the reduced BW or the center duration of open-field testing in *Slc6a1*^–/–^ KO mice (Figure 2, E–J). *Slc6a1*^+/–^ mice consistently showed slightly decreased (but not significant) rotarod performance relative to WT littermates. Although none of the differences between WT mice, treated *Slc6a1*^+/–^ mice, and untreated *Slc6a1*^+/–^ mice were significant, we observed an apparent normalization of rotarod performance in AAV9/*hSLC6A1* ICV-treated *Slc6a1*^+/–^ mice (Figure 2J). Although the ICV treatment did not affect the aggregated behavioral phenotypes we assessed in the *Slc6a1*^+/–^ mice, we did observe some adverse events (including death) across all of our neonatal ICV vector treatment groups. The JeT vector resulted in approximately 50% early mortality and some sporadic adverse effects while the MeP229 vector resulted in 17% early mortality also with some sporadic adverse effects (Supplemental Table 2). Due to the high mortality rate of mice treated with the JeT vector design, enrollment of this arm of the study was ended prematurely, and no behavioral tests were conducted on these mice.

We investigated the pattern of *hSLC6A1* transgene expression in *Slc6a1*^+/–^ mouse brain slices using in situ RNAscope. The greatest expression was seen in the neocortex and hippocampus, with less expression in the midbrain and cerebellum. The vector with the JeT promoter showed the highest transduction rates, with about 57% of cells in the neocortex, approximately 28% of cells in the hippocampus, and approximately 21% of cells in the thalamus expressing the *hSLC6A1* mRNA. The vector with the MeP229 promoter showed a transduction rate of about 35% of cells in the neocortex, approximately 22% of cells in the hippocampus, and approximately 3% of cells in the thalamus (Supplemental Figure 3). To explore how the vector-mediated expression compared with the endogenous *Slc6a1* expression pattern, we use a duplexed RNAscope assay to measure the ratio of endogenous *Slc6a1*-positive cells among the viral transduced cells in each mouse brain area (Supplemental Figure 3F). Overall, AAV9/*hSLC6A1* treatment of neonatal mice showed a strong attenuation of seizure deficits in heterozygous *Slc6a1*^+/–^ mice and both seizure and behavioral deficits in homozygous *Slc6a1*^–/–^ KO mice. These results provide early proof-of-concept evidence for possible benefits from AAV9/*hSLC6A1* treatment. However, concerning adverse effects (including mortality) were apparent with both vector designs and strongest in the JeT promoter construct.

### Intrathecal administration of AAV9/hSLC6A1 to P5 mice rescues abnormal EEG patterns in heterozygous Slc6a1^+/–^ mice and nest-building behavior in homozygous Slc6a1^–/–^ KO mice, but fails to rescue brain EEG epilepsy in Slc6a1^–/–^ KO mice.

**Figure 3:**
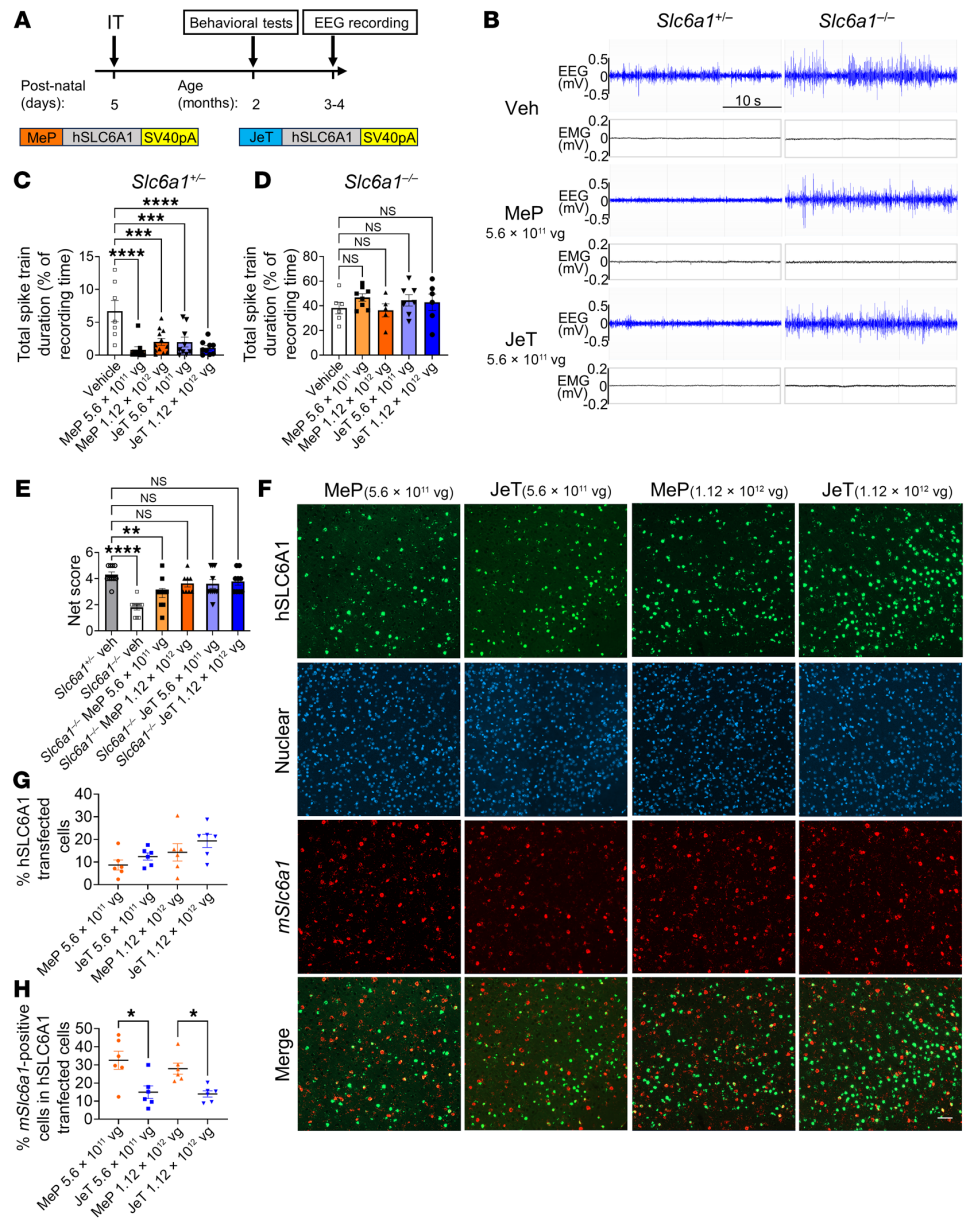
i.t. administration of AAV9/*hSLC6A1* to P5 mice rescues abnormal EEG patterns in *Slc6a1*^+/–^ mice and some cognitive behaviors in *Slc6a1*^–/–^ mice, but fails to rescue the abnormal EEG patterns in *Slc6a1*^–/–^ mice. (**A**) Experimental design of AAV9/*hSLC6A1* gene therapy via i.t. injection in P5 mice. Either vehicle, AAV9/MeP229-*hSLC6A1*, or AAV9/*JeT-hSLC6A1* were administered at doses of 5.6 × 10^11^ vg or 1.12 × 10^12^ vg. Study readouts at specified ages are listed. (**B**) Representative EEG and EMG recording examples for treated *Slc6a1*^+/–^ and *Slc6a1*^–/–^. A 10 Hz high pass filter was used to smooth the EEG signal. (**C**) Quantification of total spike train duration across a 48-hour EEG recording period (% recording time) for treated *Slc6a1*^+/–^ mice (left to right: *n* = 7, 9, 12, 9, and 9). (**D**) Quantification of total spike train duration across a 48-hour EEG recording period (% recording time) for treated *Slc6a1*^–/–^ mice (left to right: *n* = 6, 8, 5, 7, and 6). (**E**) Nest-building scores in treated *Slc6a1*^–/–^ mice compared with vehicle-treated *Slc6a1*^+/–^ mice (left to right: *n* = 10, 10, 10, 8, 10, and 9). For **C**–**E**, data are represented by the mean ± SEM. One-way ANOVA with Dunnett’s multiple-comparisons test was used for statistical analysis. (**F**) RNAscope multiplex fluorescent images showing mRNA expression of the *hSLC6A1* transgene (green) and mouse endogenous *mSlc6a1* (red) from the cortex in treated *Slc6a1*^+/–^ mouse brain sections, counterstained with DAPI (blue). Scale bar: 100 μm. (**G**) Quantification of the percentage of transduced human *SLC6A1*-positive cells in the cortex of treated *Slc6a1*^+/–^ mice. The percentage of *hSLC6A1* transduced cells are expressed as a ratio of green *hSLC6A1*-positive cells to the total number of blue nuclear DAPI positive cells (counting at least 1,000 nuclei of each sample/brain area); (**H**) Quantification of the percentage of endogenous *mSlc6a1*-positive cells in *hSLC6A1*-transduced cells in each brain region (expressed as a ratio of the double SLC6A1-positive cells [red and green] to transduced *hSLC6A1*-positive cells [green]). Data are represented as mean ± SEM. *n* = 6 per group. One-way ANOVA with Dunnett’s multiple-comparisons test was used for statistical analysis. *P < 0.05; **P < 0.01; ***P < 0.001; ****P < 0.0001.

The neonatal mouse treatment age is earlier than would be practical to model a human treatment, so we do not view the neonatal ICV approach as having direct clinical relevance. To explore the translational relevance of the JeT or MeP vector designs with AAV9, we tested both vectors by intrathecal (i.t.) lumbar puncture in heterozygous and homozygous *Slc6a1*-KO mice at P5 (Figure 3A). i.t. administration of either AAV9/MeP229-*hSLC6A1* or AAV9/JeT-*hSLC6A1* (5.6 × 10^11^ vg or 1.12 × 10^12^ vg for each) to P5 *Slc6a1*^+/–^ mice significantly reduced seizures assessed via telemetry EEG recording (Figure 3, B and C). The MeP vector at the low dose (5.6 × 10^11^ vg) and JeT vector at the high dose (1.12 × 10^12^ vg) showed the greatest normalization of the EEG patterns, equivalent to WT. However, the severe EEG seizure phenotype in *Slc6a1*^–/–^ KO mice was not rescued by either vector construct regardless of dose (Figure 3, B and D). However, both vectors normalized nest-building behavioral deficits in *Slc6a1*^–/–^ KO mice compared with vehicle-treated *Slc6a1*^+/–^ mice (Figure 3E). Neither vector significantly improved the reduced BW, fear conditioning, center duration of open field, rotarod performance, or the hind limb clasping phenotype in *Slc6a1*^–/–^ KO mice (Supplemental Figure 4), However, in rotarod tests, the JeT vector at a low dose (5.6 × 10^11^ vg) showed a trend toward increasing rotarod performance in treated *Slc6a1*^–/–^ KO mice, but this was not observed in the high-dose JeT treatment group (Supplemental Figure 4, F and G). We did not observe a dose response within the treatment groups in any of the behavioral tests, except for the nest-building tests of the MeP treatment groups (Figure 3E). Neither the MeP nor the JeT vector caused early mortality or adverse effects after i.t. administration at P5 (Supplemental Table 3). We investigated the expression pattern of *hSLC6A1* in the *Slc6a1*^+/–^ mouse brain cortex using in situ RNAscope. In general, the JeT vector showed higher transduction rates (~12% of cells at 5.6 × 10^11^ vg dose, ~19% at 1.12 × 10^12^ vg) than the MeP vector (~9% of cells at 5.6 × 10^11^ vg dose, ~14% at 1.12 × 10^12^ vg) in the brain cortex (Figure 3, F and G). We measured the ratio of endogenous *Slc6a1*-positive cells among the viral transfected cells (Figure 3H). Analyzing the MeP vector at the 5.6 × 10^11^ vg dose, approximately 33% of cells expressing the *hSLC6A1* transgene overlapped with endogenous murine *Slc6a1* (*mSlc6a1*) expression. The JeT vector at the 5.6 × 10^11^ vg dose showed only approximately 15% of cells expressing the *hSLC6A1* transgene overlapping with *mSlc6a1* expression. These results suggest that, of the 2 vector constructs, the selective expression pattern mediated by the MeP229 promoter more closely matched the endogenous *Slc6a1* expression pattern. However, the JeT promoter confers expression to a greater overall number of cells, which appears to mediate an overall more potent effect, in terms of both efficacy and adverse effects.

### i.t. administration of AAV9/hSLC6A1 to P10 or P28 Slc6a1^–/–^ KO mice showed weak or no behavioral improvements.

**Figure 4:**
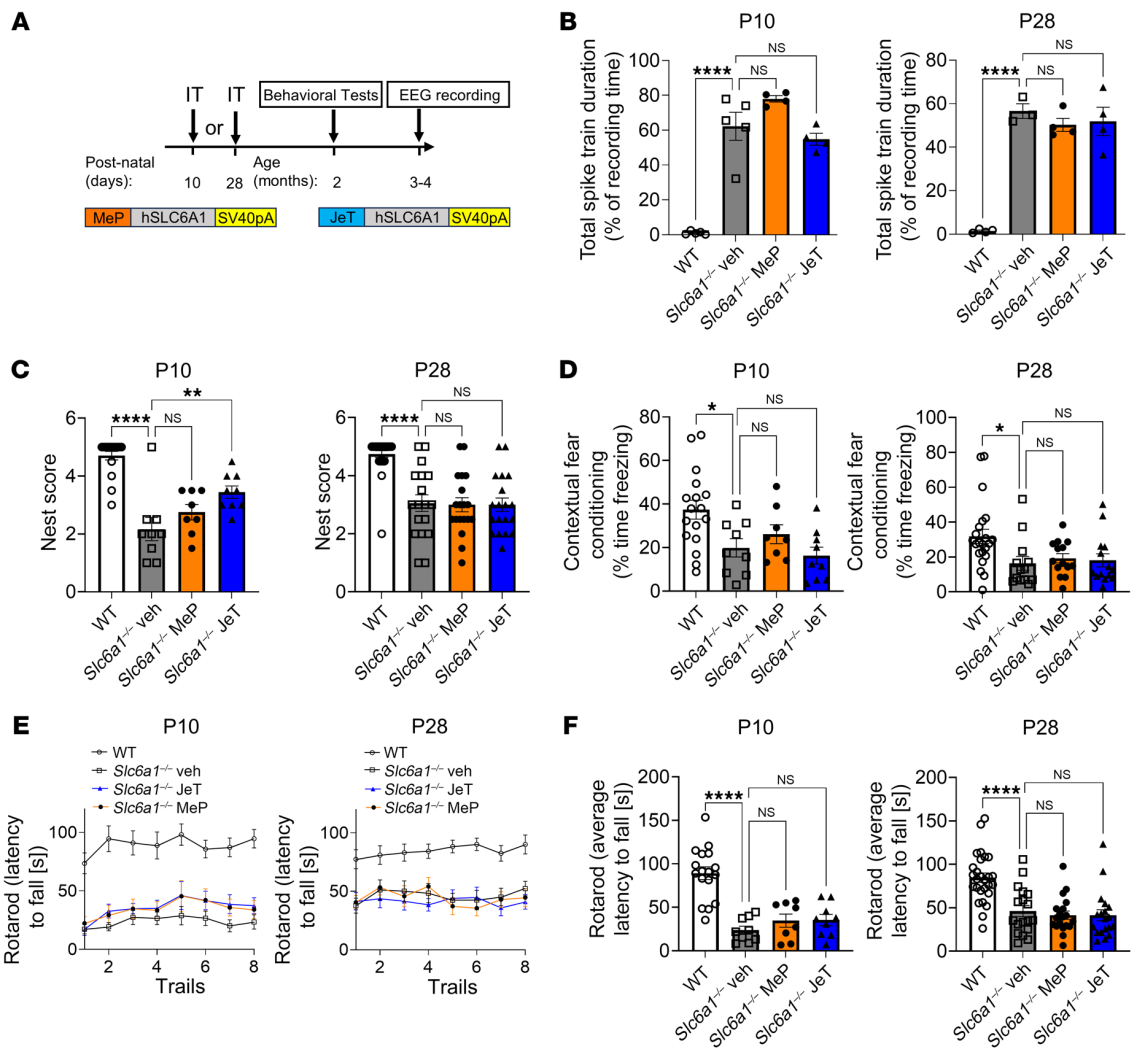
i.t. administration of AAV9/*hSLC6A1* to P10 or P28 *Slc6a1*^–/–^ KO mice showed weak or no behavioral improvements. (**A**) Experimental design of preclinical AAV9/*hSLC6A1* gene therapy via i.t. injection to P10 or P28 *Slc6a1*^–/–^ KO mice. Either vehicle, 7 × 10^11^ vg AAV9/MeP229-*hSLC6A1*, or 7.5x10^11^ vg AAV9/JET-*hSLC6A1* were administered. Study readouts at specified ages are listed. (**B**) Quantification of total spike train duration across a 48-hour EEG recording period (% recording time) (left to right: *n* = 5, 5, 4, 4, 4, 3, 4, and 4). (**C**) Nest-building scores (left to right: *n* = 17, 9, 9, 8, 27, 18, 19, and 19) (**D**) Freezing percentage of time during the contextual fear conditioning tests (left to right: *n* = 17, 9, 9, 8, 22, 15, 15, and 14). (**E**) Latency to fall (s) across 8 trials of the rotarod tests and (**F**) average latency to fall (s, average of 8 trials) of the rotarod tests (left to right: *n* = 17, 9, 9, 8, 27, 18, 19, and 19). Data are represented as means ± SEM. One-way ANOVA with Dunnett’s multiple-comparisons test was used for statistical analysis. *P < 0.05; **P < 0.01; ****P < 0.0001.

To explore the translational relevance of the JeT or MeP vector designs with AAV9, we tested both vectors by i.t. administration in *Slc6a1*^–/–^ KO mice at P10 and P28 (7.0 × 10^11^ vg for AAV9/MeP-hSLC6A1; 7.5 × 10^11^ vg for AAV9/JeT-*hSLC6A1*) (Figure 4A). Neither reduced *Slc6a1*^–/–^ KO seizures as assessed via telemetry EEG recording (Figure 4B). Mice treated at P10 via i.t. administration with the JeT vector showed improvement in nest building and modest but statistically nonsignificant improvement in rotarod performance. However, there was no apparent change for any other behavioral measures (Figure 4, C–F, and Supplemental Figure 5). We did not observe any significant improvement in any behavioral tests in P10 i.t. treated mice with the MeP vector (Figure 4, C–F, and Supplemental Figure 5). Neither the JeT nor the MeP vector P28 treated mice showed any improvement in EEG or behavior phenotypes (Figure 4, C–F, and Supplemental Figure 5). To increase the number of cells transduced at P10 via i.t. administration, we performed repeated dosing of the MeP vector in a subset of *Slc6a1*^–/–^ KO mice. Mice were given 2 subsequent i.t. injections of 1.1 × 10^12^ vg 2 hours apart (2.2 × 10^12^ vg total dose). A single i.t. injection (1.1 × 10^12^ vg total dose) group was included as a control. The highest dose (2.2 × 10^12^ vg) of the MeP vector via double i.t. administration at P10 still failed to provide any EEG pattern or behavioral benefit to *Slc6a1*^–/–^ KO mice (Supplemental Figure 6). Both AAV9/MeP-*hSLC6A1* and AAV9/JeT-*hSLC6A1* vectors delivered via i.t. administration at P10 and P28 were well tolerated even up to a dose of 2.2 × 10^12^ vg of the MeP vector at P10, with no adverse effects observed across all treated animals (Supplemental Table 3).

### AAV-PHP.eB/MeP-hSLC6A1 gene therapy initiated at P23 does not rescue abnormal EEG patterns or behavioral phenotypes in heterozygous or homozygous Slc6a1 KO mice.

**Figure 5:**
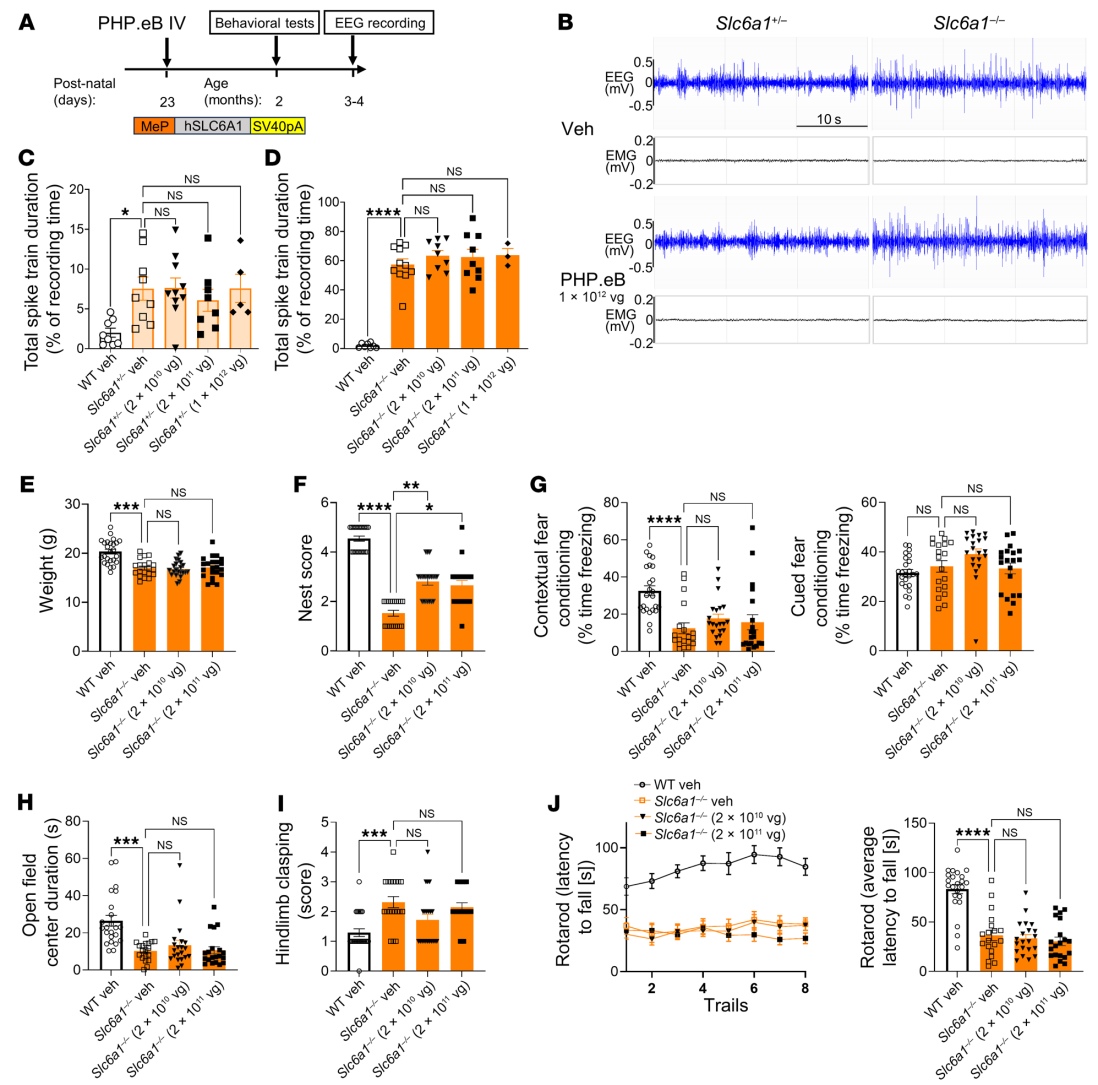
AAV-PHP.eB/MeP-*hSLC6A1* gene therapy initiated at P23 provides minimal behavior benefit and does not rescue abnormal EEG patterns in heterozygous and homozygous *Slc6a1* KO mice. (**A**) Experimental design of the i.v.-administered AAV-PHP.eB/MeP-*hSLC6A1* gene therapy approach. WT, *Slc6a1*^+/–^, or *Slc6a1*^–/–^ mice were treated at P23 with 0, 2 × 10^10^, 2 × 10^11^, or 1 × 10^12^ vg. Weight and behavioral assessments were not conducted on heterozygous mice due to their lack of a phenotype or on mice receiving the 1 × 10^12^ vg dose due to the small number of animals. Study readouts at each time point after vector administration or at specified ages are listed. (**B**) Representative EEG and EMG recording examples. A 10 Hz high pass filter was used to smooth the EEG signal. (**C**) *Slc6a1^+/–^* quantification of the total spike train duration over a 48-hour EEG recording period (% recording time) (left to right: *n* = 8, 9, 10, 8, and 5). (**D**) *Slc6a1^–/–^* quantification of total spike train duration over a 48-hour EEG recording period (% recording time) (left to right: *n* = 8, 11, 9, 9, and 3). (**E**) BWs, (**F**) nest-building scores, (**G**) contextual and cued fear conditioning freezing levels, (**H**) center time in an open field, (**I**) hind limb clasping scores, (**J**) rotarod latency to fall time. **E**–**I**, *n* = 20-25 per group. All data are represented as means ± SEM. One-way ANOVA with Dunnett’s multiple-comparisons test was used for statistical analysis. *P < 0.05; **P < 0.01; ***P < 0.001; ****P < 0.0001.

It is not clear whether the efficacy seen in the neonatal ICV treated or P5 i.t. treated mice was due to the higher transduction efficiency in neocortex and hippocampus at this age and route of administration or whether the earlier intervention was the main driver of the treatment efficacy. To begin to discriminate between these possibilities, the MeP229-*hSLC6A1* construct was packaged into the PHP.eB viral capsid/vector to be dosed into approximately P23 mice by an i.v. injection route. While PHP.eB is not directly translatable to primates, it can achieve substantially higher transduction than AAV9 following i.v. administration at an older age in mice (Supplemental Figure 7; ref. 29). We evaluated the dose-responsive safety and efficacy of PHP.eB/MeP229-*hSLC6A1* in *Slc6a1*^+/–^ and *Slc6a1*^–/–^ KO mice at P23 to determine if the disease is rescuable at that older age (Figure 5A). All doses of the PHP.eB/*hSLC6A1* vector (2 × 10^10^, 2 × 10^11^, or 1 × 10^12^ vg per mouse) failed to reduce abnormal EEG patterns in either *Slc6a1*^+/–^ or *Slc6a1*^–/–^ KO mice (Figure 5, B–D). Additionally, mice treated with the 2 × 10^10^ or 2 × 10^11^ vg doses only showed a moderate nest-building behavioral improvement (the 1 × 10^12^ vg group was not assessed for behavioral rescue) (Figure 5, E–J). Similar to P28 i.t. treatment data, P23 i.v. administration of the PHP.eB/MeP-hSLC6A1 vector failed to provide any benefit to *Slc6a1*^–/–^ KO mice. AAV-PHP.eB/MeP-*hSLC6A1* treatment at P23 was well tolerated, with no adverse effects observed in any of the i.v.-treated animals (Supplemental Table 3). We used the in situ RNAscope assay to assess the expression pattern of human *SLC6A1* transgene via AAV-PHP.eB IV administration at different doses in P23 WT mice. Compared with AAV9/MeP-*hSLC6A1* vector i.v. administration, the AAV-PHP.eB/MeP-*hSLC6A1* vector showed much greater transgene expression, evenly distributed throughout the mouse brain. The 2 × 10^11^ vg dose of PHP.eB/MeP-*hSLC6A1* vector showed an approximately 4%–5% overall cell transduction rate in the neocortex, hippocampus, and thalamus brain regions. The highest dose (1 × 10^12^ vg) showed an approximately 13% cellular transduction rate across the brain including the neocortex, hippocampus, and thalamus. Of note, compared with the neonatal ICV injections, the PHP.eB/MeP vector achieved higher transduction rates in regions outside of the neocortex and hippocampus, such as the cerebellum. We found that 20% of the PHP.eB/MeP-*hSLC6A1* transfected cells were positive for endogenous murine *Slc6a1* expression, which is similar to what we found after neonatal ICV administration with AAV9. Overall, i.v. administration of AAV-PHP.eB/MeP-*hSLC6A1* at P23 conferred more even transgene expression throughout the whole brain; however, no benefits were seen aside from a modest normalization of nest building.

### i.t. administration of AAV9/JeT-hSLC6A1 or AAV9/MeP-hSLC6A1 is safe and well tolerated in WT mice.

**Figure 6:**
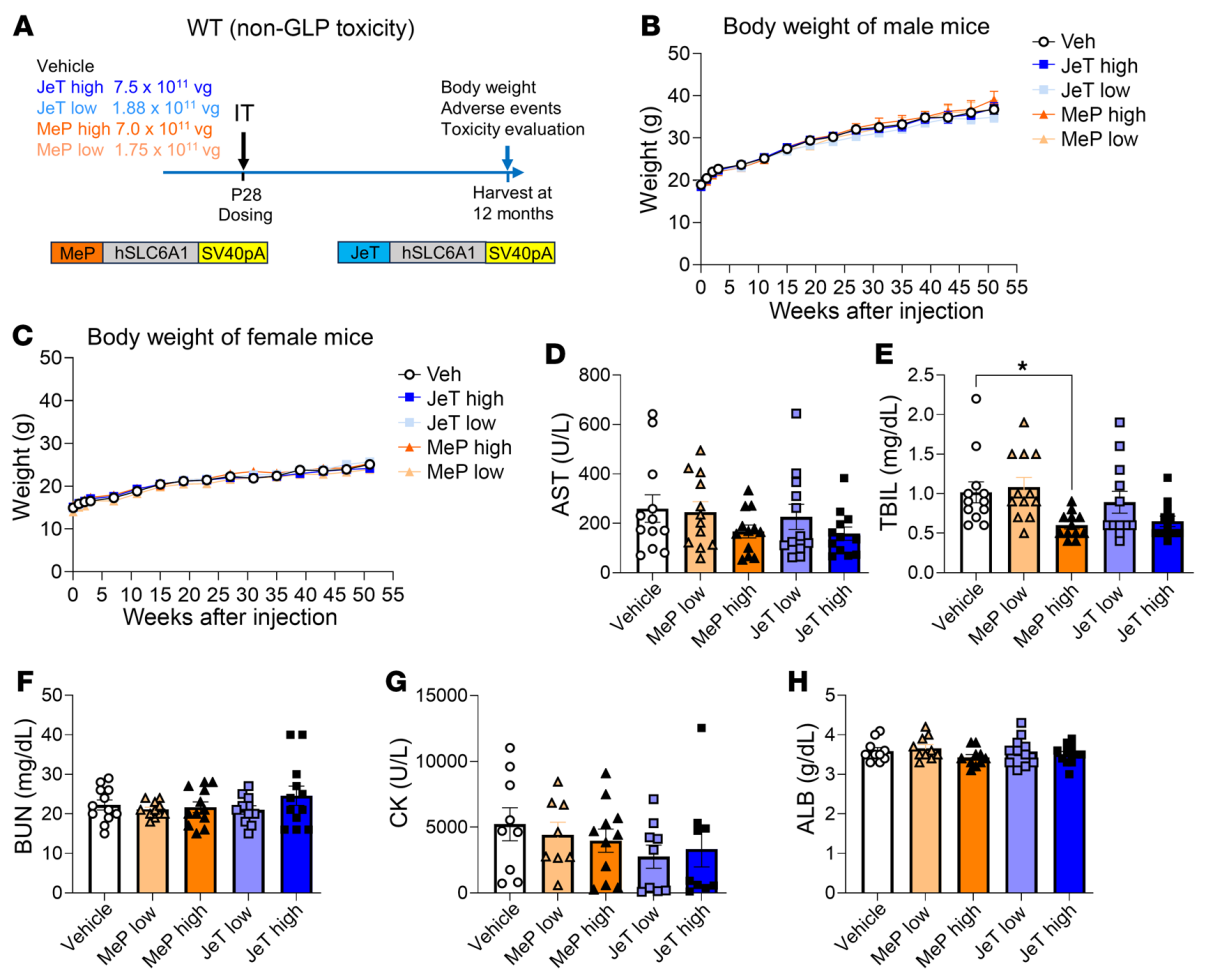
i.t. administration of either AAV9/JeT-*hSLC6A1* or AAV9/MeP-*hSLC6A1* in WT mice at P28 did not lead to any adverse effects up to a year after injection. (**A**) Experimental design of the pilot toxicology study in male and female WT C57Bl6/J mice injected with vehicle, AAV9/MeP229-*hSLC6A1* (1.75 × 10^11^ vg [low] or 7.0 × 10^11^ vg [high]), and AAV9/JeT-*hSLC6A1* (1.88 × 10^11^ vg [low] or 7.5 × 10^11^ vg [high]) via i.t. injection at P28. Study readouts at each time point after dose administration or at specified age are listed. (**B**) Longitudinal BWs of treated male mice. *n* = 6 per group. (**C**) Longitudinal BWs of treated female mice. *n* = 6 per group. (**D**–**H**) Blood chemistry tests (AST, TBIL, BUN, CK, or ALB) at 3 weeks after injection. *n* = 10–12 per group. All data are represented as means ± SEM. One-way ANOVA with Dunnett’s multiple-comparisons test was used for statistical analysis. *P < 0.05.

Overexpression of GAT1 in transgenic mice has previously been shown to result in cognitive impairment, increased susceptibility to seizures, and impaired reproduction (30, 31). In our studies, neonatal ICV administration of either AAV9/*hSLC6A1* vector design caused adverse effects in heterozygous and homozygous *Slc6a1*-KO mice, whereas administration at older ages by i.t. was well tolerated. To further evaluate the safety of the MeP and the JeT AAV9/*hSLC6A1* vectors, WT C57BL/6J mice were i.t. injected with vehicle, AAV9/JeT-*hSLC6A1* vector (1.75 × 10^11^ vg [low] or 7.0 × 10^11^ vg [high] doses), or AAV9/MeP-*hSLC6A1* vector (1.88 × 10^11^ vg [low] or 7.5 × 10^11^ vg [high] doses) at P28 in a pilot safety study (Figure 6A). The mice were monitored after injection for up to 1 year for BW, survival, body condition, blood chemistry, and comprehensive postmortem histopathology. There were no significant differences in BW between groups in male or female mice at any point of assessment and any dose tested (Figure 6, B and C). Serum from mice taken at 3 weeks after dosing was analyzed for blood biochemistry, including total bilirubin (TBIL), blood urea nitrogen (BUN), albumin (ALB), creatine kinase (CK), and aspartate aminotransferase (AST). None of the above showed significant changes induced by either vector, with the exception of a reduction in bilirubin levels observed in the MeP high-dose–treated WT mice (Figure 6, D–H). Likewise, there were no treatment-related signs of body condition change across the duration of the study in any group. Given the findings from neonatal treatment that the JeT construct posed a greater safety concern (Supplemental Table 2), tissues from all JeT-injected WT mice were evaluated for histopathology abnormalities. We have included all findings in Supplemental Table 3 and Supplemental Data Set 1, which, in summary, only identified findings typical in aged mice, with no relation to treatment. In another pilot toxicity study, WT C57BL/6J mice were i.t. injected with vehicle, AAV9/JeT-*hSLC6A1* vector, or AAV9/*MeP-hSLC6A1* vector at an earlier age (P10). The mice were monitored after injection for up to 6 months for BW, survival, body condition, and histopathology abnormalities (JeT only) (Supplemental Figure 8A and Supplemental Data Set 1). The P10 i.t. administration also did not affect any of the parameters listed in WT mice (Supplemental Figure 8, B and C). Single-cell RNA-Seq was used to assess the JeT vector-induced expression of the human *SLC6A1* transgene compared with the endogenous murine *Slc6a1* levels in WT mice from this P10 i.t. safety study. The uniform manifold approximation and projection (UMAP) plot displays the results of unbiased clustering of 27,375 single-cell nuclei from mouse brain cortex tissue following high-dose (7.5 × 10^11^ vg) i.t. treatment of the AAV9/JeT-*hSLC6A1* vector at P10 (Supplemental Figure 8D). The mouse endogenous *Slc6a1* mRNA expression is selectively enriched in the clusters of GABAergic inhibitory neurons, astrocytes, and oligodendrocyte precursor cells (Supplemental Figure 8E and Supplemental Data Set 2). The JeT promoter–driven human *SLC6A1* transgene mRNA expression was more evenly distributed throughout all the clusters, consistent with the nonspecific nature of the JeT promoter (Supplemental Figure 8F and Supplemental Data Set 2). There were 57 cells analyzed that had overlapping expression of human and mouse *Slc6a1*, allowing for direct comparison of *SLC6A1* expression levels in those cells. Of the 57 overlapping cells, compared with the endogenous mouse *Slc6a1* expression level, 40 cells had the same level of mRNA expression, in 15 cells, the expression of JeT-*hSLC6A1* was slightly higher, and in 2 cells, the JeT-*hSLC6A1* expression was lower (Supplemental Figure 8G). Whenever the human and murine *SLC6A1* levels differed, the fold change was relatively small. Overall, these results demonstrate that the JeT promoter drives mRNA expression in the neocortex, with approximately 20% of cells that normally express *Slc6a1*. This leads to about 80% of transduced cells gaining expression of *SLC6A1* where it is not normally expressed.

## Discussion

Recombinant AAV9-mediated gene therapy has been widely applied in preclinical and clinical research for the treatment of CNS disorders, often using an i.t. administration route (13, 15). In this study, we have designed an AAV-based treatment strategy that delivers the codon-optimized human *SLC6A1* gene under different regulatory promoters (MeP or JeT) to a mouse model of *SLC6A1*-related disorders. We demonstrated AAV9/*hSLC6A1* gene therapy has potential as a treatment for *SLC6A1*-related disorders, when administered at early development ages.

At the beginning of our study, there was uncertainty regarding the suitability of available *SLC6A1* mouse models and how different *SLC6A1* variant alleles might contribute to distinct disease phenotypes. Upon assessing *SLC6A1* animal models, we found that the heterozygous *Slc6a1*^A288V/+^ KI mice and the heterozygous *Slc6a1*^S295L/+^ KI mice showed abnormal EEG phenotypes identical to those of the heterozygous *Slc6a1*^+/–^ KO mice. Furthermore, as a partial loss-of-function variant, the homozygous *Slc6a1*^A288V/A288V^ KI mice showed somewhat milder behavioral phenotypes than the homozygous *Slc6a1*^S295L/S295L^ (complete loss of function) and the homozygous *Slc6a1*^–/–^ null KO mice (Supplemental Table 1). The majority of evidence supports that haploinsufficiency or loss of function of SLC6A1 primarily underlies the phenotypes of *Slc6a1* mouse models and human clinical symptoms, rather than gain-of-function or dominant-negative effects. Therefore, the heterozygous and homozygous *Slc6a1* KO models are suitable to test an AAV9 gene replacement approach. Additionally, the milder cognitive behavioral and motor phenotypes observed in *Slc6a1* A288V homozygous mice suggest that the functional dosage of *Slc6a1* critically underlies the pathogenic processes associated with *SLC6A1*. The *SLC6A1* haploinsufficiency concept is also supported by a recent study that identified more than 200 loss-of-function *SLC6A1* variants from patients with ASD without overt epileptic phenotypes (32). The human clinical phenotype includes 2 common domains of symptoms: epilepsy and neuropsychological features affecting cognition and behaviors. EEG abnormalities in human patients are most commonly generalized epileptiform discharges. When available, EEG recordings with seizure semiology data have identified (atypical) absence, atonic, and myoclonic seizures as the dominant types of seizures seen in patients (1). Although the technical limitations of the 2 electrode EEG sensors used in this study prevented us from defining the seizure semiology in *SLC6A1* mice, we were able to robustly identify polyspike-wave discharges paired with a cessation of muscle activity, typical of absence seizures. These findings suggest that the *SLC6A1* mouse models we tested not only have similar epileptic phenotypes to each other but are characteristically similar to the prevalent epilepsy phenotypes in humans. We concluded that the *Slc6a1*^–/+^ heterozygous mice phenotypically model the clinical epilepsy phenotype of human patients but do not strongly represent the cognitive-behavioral phenotypes observed in human patients (Figure 1). On the other hand, the homozygous *Slc6a1*^–/–^ KO mice phenotypically model severe cognitive-behavioral features seen in human patients, including learning and memory deficits and anxious behaviors, as well as developmental and motor disorders. However, the epilepsy phenotype is much more severe in the homozygous KO mice than in the heterozygous mice (Figure 1, A–C). Our studies evaluated treatment in both models, in an attempt to comprehensively evaluate both behavioral and epilepsy symptoms across the 2 models.

GABA, acting as the predominant inhibitory neurotransmitter, also plays a crucial role in governing the migration and maturation processes of newly formed neurons throughout the development of the CNS (33). The regulation of GABA reuptake levels through the functioning of the *SLC6A1* gene may be implicated throughout critical periods from the embryonic period through adolescence. Therefore, AAV9/*hSLC6A1* gene transfer may variably impact different disease phenotypes if AAV9/*hSLC6A1* is delivered after critical periods of CNS development (34). In our studies, we selected multiple treatment ages (P1, P5, P10, P23, and P28) in an effort to explore the impact of treatment at different developmental ages. Regarding the abnormal EEG phenotypes in heterozygous *Slc6a1*^+/–^ mice, both neonatal P1 ICV and P5 i.t. treated mice displayed therapeutic benefit (substantially normalized EEG epilepsy patterns) from AAV9/MeP-*hSLC6A1* or AAV9/JeT-*hSLC6A1* treatment (Figure 2). This benefit was not seen in the older P23 treatment groups (P10 and P28 treatment was not tested in heterozygous *Slc6a1*^+/–^ mice) (Figure 5). For homozygous *Slc6a1*^–/–^ KO mice (which have more severe EEG phenotypes), neonatal ICV administration effectively rescued the abnormal EEG patterns (Figure 2). However, at P5 and older, the AAV9/*hSLC6A1* treatment failed to show any benefit on the EEG pattern. One might infer that in homozygous KO mice, the pathogenic epileptic circuits developed during the early development period are different from those in heterozygous mice. Our studies indicate that it is ineffective to rescue them by gene replacement outside of this period. For behavioral phenotypes in homozygous KO mice, multiple behaviors were ameliorated with gene-therapy treatment from P1 to P5. However, in older KO mice (P10 and P23 treatments), efficacy is much lower, with only nest-building deficiency being ameliorated (Supplemental Table 4). Considering the different therapeutic response between *Slc6a1*^+/–^ and *Slc6a1*^–/–^ mice, our results suggest that for the epilepsy phenotype, the complete loss of SLC6A1 function creates more severe and potentially irreversible changes in brain circuitry beyond very early mouse postnatal periods. In contrast, the presence of approximately 50% functional SLC6A1 through early development made the *Slc6a1*^+/–^ mice receptive to treatment and phenotypic reversal was possible up to at least P5. An important caveat is that gene-transfer efficiency was also higher in younger mice. Ultimately, a definitive answer to this question of timing and phenotypic “reversibility” may require the use of genetic reconstitution of SLC6A1 in mouse models, such as was done for Rett syndrome (35). Until then, our results warrant caution in evaluating other therapeutic candidates for *SLC6A1*-related disorders in homozygous KO mice outside of neonatal administration: their complete absence of functional *Slc6a1* does not match the human haploinsufficiency condition, and aspects of their disease may be too severe to rescue. In the absence of further orthogonal data on the topic, our current conclusion is that heterozygous mice are likely the best model to evaluate the effectiveness of any potential therapeutics for *SLC6A1*-related disorders.

The *SLC6A1* gene is primarily expressed in the CNS of mammals and predominantly expressed in GABAergic inhibitory neurons, although it can also be found in glia cells (36, 37). In our 2 AAV9/*hSLC6A1* vector designs, both JeT and MeP229 promoters will express human *SLC6A1* outside of cells that typically express endogenous *Slc6a1* (Supplemental Figure 9). This raises a theoretical question of whether the ectopic overexpression of *SLC6A1* outside of cells that normally express *SLC6A1* could be helpful or harmful. From an efficacy perspective, the ectopic expression of *hSLC6A1* in excitatory neurons or other cell types may have global effects in restoring extracellular GABA levels through the GABA uptake function of SLC6A1 protein. However, it may also influence excitatory transmission and activity. Both excitatory and inhibitory activity contribute to the underlying mechanisms of the epileptic and behavioral phenotypes in the *Slc6a1* mouse models. These dual effects on both excitatory and inhibitory circuits should be considered in efficacy studies. The treatment window may also be affected or altered by the balance between the harmful and beneficial effects of ectopic expression. Future vector designs that restrict ectopic expression of *hSLC6A1* may help address these questions, but this is beyond the scope of our current study. Based on in situ RNAscope analysis and single-cell RNA-Seq data, the JeT AAV9/*hSLC6A1* vector shows a much broader expression in the mouse brain compared with MeP-*SLC6A1*. Almost approximately 80% of JeT-*hSLC6A1*-expressed cells do not overlap with the *mSlc6a1*-positive cells. However, a positive aspect is that the transcription levels of human JeT-expressed *SLC6A1* are equal to or slightly higher than the expression levels of the endogenous mouse *Slc6a1* following i.t. injection (Supplemental Figure 8). In terms of efficacy, the MeP vector demonstrated either better or equal efficacy compared with the JeT vector in most assessments. This was particularly evident in the early stages of development, as observed in neonatal and P5 treated mice. Our RNAscope data revealed that both the MeP and JeT vectors transfected a similar percentage of brain cortex cells that normally express *Slc6a1* (Figure 3). From a safety perspective, the neonatal ICV treatment group exhibited a high degree of adverse effects, particularly with the JeT promoter but also to a lesser extent with the MeP promoter. While we can’t rule out general toxicity of AAV9 at this age and route, we conclude that this toxicity is most likely due to expression of the *SLC6A1* transgene due to the following: (a) The effects seemed to be somewhat promoter dependent, even when the 2 SLC6A1 vectors were administered at the same dose. (b) While we were not able to determine the cause of death of mice that died prematurely, limited histological examination didn’t show signs of neurodegeneration (data not shown), and the appearance of convulsive seizures in some mice is consistent with the notion of a GABA-related excitatory/inhibitory imbalance. (c) In other studies conducted by our group using similarly manufactured AAV, we have not observed this type of toxicity from AAV9 vectors carrying other transgenes (for example, an AAV9/FIG4 vector injected at a higher dose of 5.4E11 vg ICV at P1; ref. 38). We would hypothesize that a lower dose would be tolerated better, but this wasn’t tested. In contrast, the ectopic overexpression of human *SLC6A1* by MeP vectors appeared to be well tolerated at P5 or older age treatment groups via i.t. injection. The JeT vector showed higher adverse events in response to earlier age treatment groups, including neonatal and P5, but at P10 and P28 ages, the JeT vector was well tolerated in both treated WT and KO mice. Based on these data, the JeT vector exhibited notably greater safety concerns, suggesting that either the overall higher expression or the expression in non–*mSlc6a1*-expressing cells is detrimental. Since the JeT vector did not provide additional efficacy, it may be advisable to use a more restrictive promoter.

While neonatal ICV injection in mice may not be directly translationally relevant due to differences in developmental ages between mice and humans, the observed benefits to treated mice still provide proof of concept that AAV9 gene replacement therapy can normalize both epilepsy and cognitive behavioral phenotypes in *Slc6a1* heterozygous and homozygous mice. Considering the challenges imposed by the mouse model phenotypes in relation to the human condition, there are multiple implications and caveats stemming from our results. As the *Slc6a1*^+/–^ model shows minimal to no behavioral abnormalities, our experiments in that model could not address whether our gene-therapy approach has an impact on the behavioral aspects of the disease. Our results following neonatal ICV treatment of *Slc6a1*^–/–^ mice and the nest-building results from P5 treatment of *Slc6a1*^–/–^ mice suggest that the vector can rescue at least some aspects of behavior, at least at this early age, but we may not have appropriate models to answer the specific question of behavioral rescue at later ages. In terms of the age dependence of the treatment in heterozygous mice, the gene-therapy treatment was effective at P5 and ineffective at P23 (with PHP.eB). However, it remains possible that a P23 rescue is possible with higher gene-transfer efficiency. Future studies will need to fine tune that intervention window. When considering the equivalent age in humans, an important caveat is that there is an expected time course of at least 2 weeks before the transgene expression reaches maximal levels. At this early age in a mouse, over the course of 2 weeks the brain undergoes very rapid growth through multiple phases of critical brain development. In a human, that same period of brain growth and development stretches over years, where (relatively speaking) the transgene expression will turn on very rapidly. Thus, we conclude that early intervention is important to maximize efficacy in this gene-therapy approach for *SLC6A1*, but extreme caution should be taken trying to extrapolate equivalent treatment windows in a human.

## Methods

### Sex as a biological variable.

Our study examined male and female animals, and similar findings are reported for both sexes.

### Viral vector design, development, and vector preparation.

We designed and developed the MeP229-*hSLC6A1*-SV40pA and JeT-*hSLC6A1*-SV40pA plasmids (Figure 2A). The transgene plasmid consists of a human *SLC6A1* codon-optimized DNA coding sequence of 1800 bp (*hSLC6A1opt*) between a 229 bp MeP229 promoter or a 164 bp JeT promoter and a 123 bp SV40pA polyadenylation signal. The MeP229 and JeT plasmids were packaged into scAAV9 vectors. The scAAV9 vectors were produced at the University of North Carolina at Chapel Hill (UNC) Vector Core or University of Texas Southwestern (UTSW) Vector Core, and titered by qPCR.

### SLC6A1 animals.

*Slc6a1*^–/–^ KO mice (B6.129S1-*Slc6a1*tm1Lst/Mmucd, RRID: MMRRC_000426-UCD) were from the Mutant Mouse Resource and Research Center (MMRRC) at University of California at Davis (originally donated by Henry Lester, California Institute of Technology, Pasadena, California, USA), *Slc6a1*^S295L/S295L^ mice were from Shanghai Model Organisms (C57BL/6-*Slc6a1*em2(S295L) Smoc, catalog number NM-KI-190014. *Slc6a1*^A288V/A288V^ mice were obtained through a collaboration with Jing Qiong Kang at Vanderbilt University (Nashville, Tennessee, USA). All the mice used in this study were received by our lab on the C57BL6/J background. Each strain was then backcrossed to WT C57BL6/J mice (purchased from the Jackson Laboratory) by our lab for at least 4 generations prior to use in any experimental studies. Breeding strategies for these mice consisted of heterozygous male mice crossed with heterozygous female mice. The exception to the HET × HET breeding strategy was in generating *Slc6a1*^–/–^ and *Slc6a1*^+/–^ mice for the ICV studies. Here, a *Slc6a1*^–/–^ male was crossed with *Slc6a1*^+/–^ female mice. A harem breeding scheme was used throughout; 1 male mouse was housed permanently with 2 female mice.

### Efficacy study design.

The goal of our study was to develop and characterize a potential AAV9 gene-replacement therapy for human *SLC6A1*-related disorders. We first characterized 3 animal preclinical models of *SLC6A1*-related disorders, recapitulating core features pathologically and phenotypically observed in human *SLC6A1*-related disorders. The heterozygous *Slc6a1*^+/–^ KO and homozygous *Slc6a1*^–/–^ KO mice were chosen as the primary models for our gene-replacement studies. Two AAV9 vectors were designed, carrying human *SLC6A1* driven by either the weak universal promoter (JeT) or the predominantly neuronal promoter (MeP229). An initial proof-of-concept study was carried out in neonatal *Slc6a1*^+/–^ and homozygous *Slc6a1*^–/–^ KO mice by ICV administration. A larger and more comprehensive age- and dose-response study was then carried out in P5, P10, P23, and P28 mice by i.t. lumbar puncture administration or i.v. administration (P23 only, with a PHP.eB/MeP vector). Animal numbers used in each experiment are provided in the corresponding figure legends. We included approximately equal numbers of male and female mice in our study. Mice of each genotype were randomly allocated to treatment or control groups within each study. All outcome tests were conducted and analyzed in a blinded manner. In our analysis, sex did not affect the significance of any of the data presented in this study. We combined the data from both sexes to present the figures more concisely.

### AAV dosing for efficacy studies.

Mixed litters of WT, *Slc6a1*^+/–^ KO, and *Slc6a1*^–/–^ KO mice were randomized into treatment groups for each study described throughout the results section. For neonatal ICV injections, cryoanesthetized mouse pups received bilateral bolus injections of vehicle or AAV into the lateral ventricles, 1.5 μL per hemisphere. For i.t. injections, restrained but unanesthetized mice received a single bolus injection of vehicle or AAV, into the L4/L5 region of the lumbar spinal cord, in a volume of 5–8 μL. For the experiments described in Supplemental Figure 6, a single i.t. injection of 10 μL or 2 sequential i.t. injections of 10 μL each were administered to the mice 2 hours apart. For i.v. injections, restrained mice received a bolus injection of vehicle or AAV into the tail vein in a volume of 20 μL.

### EEG and analysis.

DSI PhysioTel HD-X02 implantable telemetry sensors were used throughout this study and implanted subcutaneously in WT, *Slc6a1*^+/–^, and *Slc6a1*^–/–^ KO mice using the Data Sciences International (DSI) manual guidelines for telemetry implantation surgery. Mice used for EEG telemetry recordings were 3–4 months old, and we used both male and female mice. Following surgeries, mice recovered for about 2 weeks. Video monitoring with synchronized EEG and electromyography (EMG) recordings was conducted continuously for 48 hours using DSI PhysioTelwireless receives and Ponemah software at the Neuro Models Core of UTSW. A 10 Hz high pass filter was used to smooth the EEG signal for seizure detection. The seizure detection (Spike Train) analysis module of NeuroScore Software scans the EEG signal to detect seizure activity with the parameters (absolute threshold: 200 μV; max spike interval: 1.5 seconds; min no. spikes: 3). For the EEG data, since all the WT mice were from the same C57BL6/J background, and they all showed identical EEG patterns, we collapsed all of the WT littermates from all 3 mouse model lines into a single group.

### Behavioral tests.

Behavioral testing of all *Slc6a1* KO mice (WT, *Slc6a1*^+/–^, and *Slc6a1*^–/–^), S295L (WT, *Slc6a1*^S295L/+^, and *Slc6a1*^S295L/S295L^) and A288V (WT, *Slc6a1*^A288V/+^, and *Slc6a1*^A288V/A288V^) mice was carried out in mice at approximately 2 months old, in males and females. All mice were put through the same behavioral battery of tests at 3 months old. Testing was performed in the following order: nest building, open field, social interaction, marble burying, rotarod, and fear conditioning. The mice were not retested on any of the aforementioned tests; however, they were trained (as is standard) for rotarod and fear conditioning. Training was conducted before the testing date for each, but after all other evaluations. The mice were then allowed to rest for at least 2 weeks before surgery was performed to implant the EEG/EMG sensor at 4 months old. Behavioral tests were carried out by investigators blinded to the genotype and/or treatment of the mice. Fear conditioning, rotarod and open-field behavioral tests were performed at UTSW Rodent Behavior Core.

### Fear conditioning.

Fear conditioning is measured in boxes equipped with a metal grid floor connected to a scrambled shock generator (Med Associates Inc.). For training, mice were individually placed in the chamber. After 2 minutes, the mice received 3 tone-shock pairings (30 second white noise, 80 dB tone coterminated with a 2 second, 0.5 mA foot shock, 1 minute intertrial interval). The following day, memory of the context was measured by placing the mice into the same chambers and freezing was measured automatically by the Med Associates software (Video Freeze Version 2.7.3.0) for 5 minutes. Forty-eight hours after training, memory for the white noise cue was measured by placing the mice in a box with altered floors and walls, different lighting, and a vanilla smell. Freezing was measured for 3 minutes, then the noise cue was turned on for an additional 3 minutes and freezing was measured.

### Rotarod.

Mice were placed onto a stationary rod (Columbus Instruments) and then the rod was started at 4 rpm. The rod was 3 cm diameter and made from a textured hard plastic. Any mice that fell off the stationary rod or the slow-moving rod were replaced until they could stay on for 2–3 seconds. The acceleration began from 4 to 40 rpm over 5 minutes (acceleration increases 0.5 rpm every 4 seconds). The time until the mouse fell from the rod was recorded. Any mouse that held on to the rotating rod for a full turn was scored as if it fell on the turn. Each mouse received 4 trials a day (with a minimum of 20 minutes intertrial interval) for 2 days.

### Open field.

Mice were placed in the periphery (placed along a wall, but in the center of the wall) of a novel plastic open-field environment (44 cm × 44 cm, walls 30 cm high) in a dimly lit room (approximately 60 lux) and allowed to explore for 10 minutes. The animals were monitored from above by a video camera connected to a computer running video-tracking software (Ethovision, Noldus) to determine the time, distance moved, and number of entries into 2 areas: the periphery (5 cm from the walls) and the center (14 cm × 14cm). The open-field arenas were wiped with Coverage Plus NPD Cleaner Disinfectant (STERIS catalog 638708) and allowed to dry between mice.

### Nest building.

The nest-building test has been described in detail previously (39).

### Hindlimb clasping.

Mice were suspended just above the bedding of an empty cage by holding the mouse about 1/2 inch from the base of the tail. The front paws were allowed to touch the bedding in the cage but unable to bear weight. The mouse was suspended for a total of 30 seconds to evaluate hind limb clasping score with scores range from 0 to 5: 0 = no fist making/clasping of paws, no retraction of either hind limb toward body. Limps stay splayed/extended for the entire 30 seconds; 1 = fist making/clasping, and/or quick retraction with immediate reextension of one hind limb; 2 = fist making/clasping, and quick retraction with immediate reextension of both hind limbs (alternating retraction of limbs); 3 = fist making/clasping, alternating or simultaneous retraction of limbs, no extension of retracted limb (retraction hold) within 1–5 seconds; 4 = fist making/clasping, alternating or simultaneous retraction of limbs, no extension of retracted limb (retraction hold) of 1 limb for longer than 5 continuous seconds; 5 = fist making/clasping, alternating or simultaneous retraction of limbs, no extension of retracted limb (retraction hold) of both limbs for longer than 5 continuous seconds. The final score is determined by averaging the results of multiple tests to enhance accuracy.

### Pilot safety study in WT BL/6J mice.

The pilot safety studies were designed to identify any short-term or long-term safety issues associated with the AAV9/*SLC6A1* treatment. They were not conducted in strict compliance with good laboratory practice (GLP). The non-GLP safety study was initiated in WT C57BL/6J mice at P28. Six males and 6 females per cohort were injected i.t. with 5 μL of vehicle, low and high doses of AAV9/MeP229, or JeT *SLC6A1* vector. Treated mice were monitored for BW, survival, and body condition, and assessed for signs of adverse effects weekly for the first 4 weeks and monthly thereafter, up to 1 year after injection. Blood samples were collected at 3 weeks and at 12 months after injection for serological assessment of hepatic and renal function (TBIL, ALT, AST, BUN, and CK). A comprehensive necropsy was conducted at 12 months after injection for histopathology. In another non-GLP toxicity study, WT C57BL/6J mice were i.t. injected with 5 μL of vehicle, low and high doses of AAV9/MeP229, or JeT *SLC6A1* vector at P10. Treated mice were monitored for BW, survival, and body condition and assessed for signs of adverse effects for up to 6 months after injection. Histological assessment of all major organs and tissues of all study mice was carried out by a veterinary pathologist who was blinded to treatment.

### RNAscope staining and image analysis.

Animals were perfused for 5 minutes with 1× PBS at a rate of 3.5 mL/min. The tissues were then harvested and fixed in 10% neutral-buffered formalin (NBF) for 24 hours before being transferred to 70% ethanol. Subsequently, the tissues were processed, embedded in paraffin, and cut into 5 μm sections. Separate sections were utilized for RNAscope to detect human transgene *hSLC6A1* mRNA or mouse endogenous *Slc6a1* mRNA using custom-made *hSLC6A1*-Codon-C1 probe and mouse Mm-Slc6a1-C3 probe, following the RNAscope Multiplex Fluorescent Reagent Kit, version 2, protocol, nuclear staining with DAPI dye. All stained brain slides, with 1 section for each animal, were captured using the Zeiss Axioscan 7 slide scanner. The cortex, hippocampus, thalamus, and cerebellum regions of the brain were selected for cell count analysis, with at least 1,000 nuclei counted in each brain area per sample.

### Analyses of single-cell transcriptomes.

A single nuclei suspension from the mouse brain cortex was prepared as previously described (40). A concentration of 1,000 nuclei/μL was submitted for 10X Genomics sequencing at the UTSW Next Generation Sequencing Core. Cell Ranger 5.0.1 (10x Genomics, https://www.10xgenomics.com/) was used to process the raw sequencing data. BCL files were converted to FASTQ files and aligned to customized mouse (mm10) reference transcriptome generated with cellranger mkref. Transcript counts of each cell were quantified using unique molecular identifier and valid cell barcode. The gene expression matrix from Cell Ranger was used as input to the Seurat R package (version 4.0.5) for downstream analysis. Cells with fewer than 250 genes per cell and high mitochondrial gene content were filtered out. The global-scaling normalization method LogNormalize was used for normalization. A subset of genes exhibiting high variation across the single cells was determined. The highly variable genes were calculated using the FindVariableFeatures module. The shared nearest neighbor (SNN) graph was constructed with the FindNeighbors module by determination of the k-nearest neighbors of each cell. The clusters were then identified by optimization of SNN modularity using the FindClusters module. We obtained 25 clusters with a resolution of 0.6. Clusters were merged and labeled to 12 clusters on the basis of known gene markers specific to various cell types. Differential expression analysis, clusters visualization, and plotting were all performed with Seurat.

### Study approval.

All animal experiments were reviewed and approved by the UTSW Institutional Animal Care and Use Committee.

### Statistics.

All quantitative data in the figures are presented as mean ± SEM, analyzed using GraphPad Prism Software (version 9.4.1). Based on the ROUT test, only 3 outliers in Figure 4D were excluded from the calculation (see Supporting Data Values). Differences between data sets were carried out using 1-way ANOVA with Dunnett’s multiple-comparisons test or 2-way ANOVA with Šidák’s multiple-comparisons test as indicated in each figure legend. *P* < 0.05 was considered significant for all statistical analyses.

### Data availability.

All data associated with this study are available in the main text or the supplemental materials, and additional study details are available from the authors upon request. Any plasmids, vectors, or animal models used in this study are available upon request through a material transfer agreement. The single-cell RNA-Seq data are available in the NCBI’s Gene Expression Omnibus database (GEO GSE278732). Values for all data points in graphs are reported in the Supporting Data Values file.

## Author contributions

WG, MR, FS, and SJG conceptualized the study, WG, MR, FS, and YH performed experiments and data analysis. ZY and CX helped with bioinformatics analysis for single-cell RNA-Seq. SJG supervised all activities of the study. WG, MR, and SJG wrote the original manuscript draft. WG, MR, FS, YH, ZY, CX, and SJG reviewed the manuscript. The order of co–first authors was established based on equal overall contributions to the project, with WG listed first due to his lead role in creating the original manuscript draft.

## Supplementary Material

Supplemental data

Supplemental data set 1

Supplemental data set 2

Supplemental video 1

Supporting data values

## Version 1 11/26/2024

In-Press Preview

## Version 2 02/03/2025

Electronic publication
