# Supplementary material for: AAV9/SLC6A1 gene therapy rescues abnormal EEG patterns and cognitive behavioral deficiencies in Slc6a1–/– mice
Source: J Clin Invest. 2024 Nov 26;135(3):e182235. doi: 10.1172/JCI182235 (PMC11785923; doi:10.1172/JCI182235)
Supplement: Supplemental data [file jci-135-182235-s037.pdf]

Supplementary data for

AAV9/*SLC6A1* gene therapy rescues abnormal EEG patterns and cognitive behavioral deficiencies in *Slc6a1*-/- mice

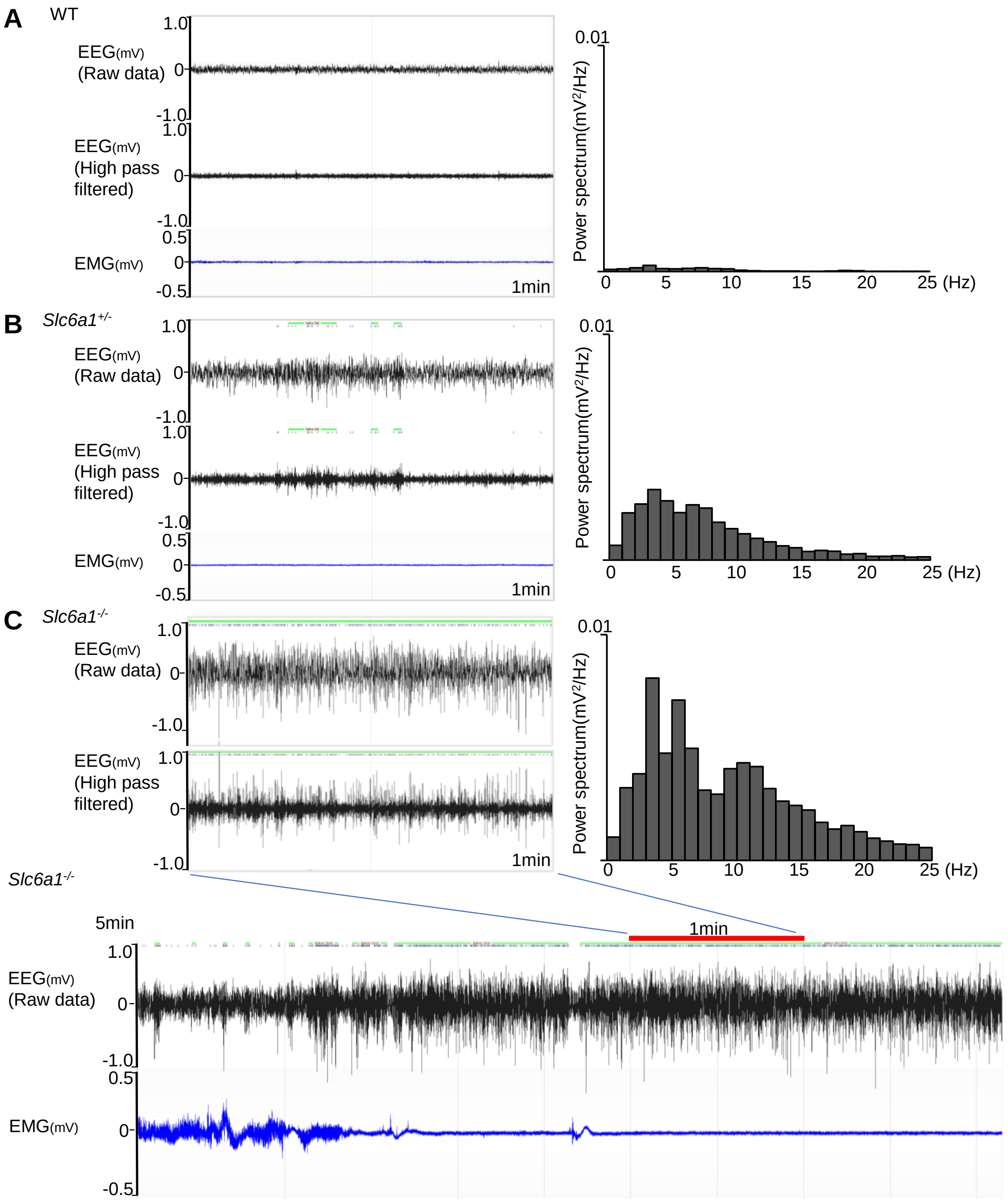

Supplemental Figure 1.The *Slc6a1*$^{+/-}$ and *Slc6a1*$^{-/-}$ mice exhibited abundant ~3-7 Hz polyspike train activities. A 10Hz high pass filter was used to smooth the EEG signal for polyspike train detection (related to Figure 1). A) Representative 1min WT EEG raw trace (Left: top), smoothed EEG trace by 10Hz high pass filter for spike detection(Left: middle) and EMG raw trace (Left: bottom). WT EEG power spectral density plot from 1min raw trace(Right). B) Representative 1min *Slc6a1*$^{+/-}$ EEG raw trace (Left: top), smoothed EEG trace by 10Hz high pass filter for spike detection (Left: middle) and EMG raw trace (Left: bottom). *Slc6a1*$^{+/-}$ EEG power spectral density plot from 1 min raw trace(Right). C) The upper panel, representative 1min Slc6a1$^{-/-}$ EEG raw trace (Left: top) and smoothed EEG trace by 10Hz high pass filter for spike detection(Left: bottom). Slc6a1$^{-/-}$ EEG power spectral density plot from 1 min raw trace(Right). The lower panel, the longer 5 min *Slc6a1*$^{-/-}$ EEG raw trace with multiple poly spike trains (top) and EMG raw trace(bottom). The solid green line indicates each individual spike train.

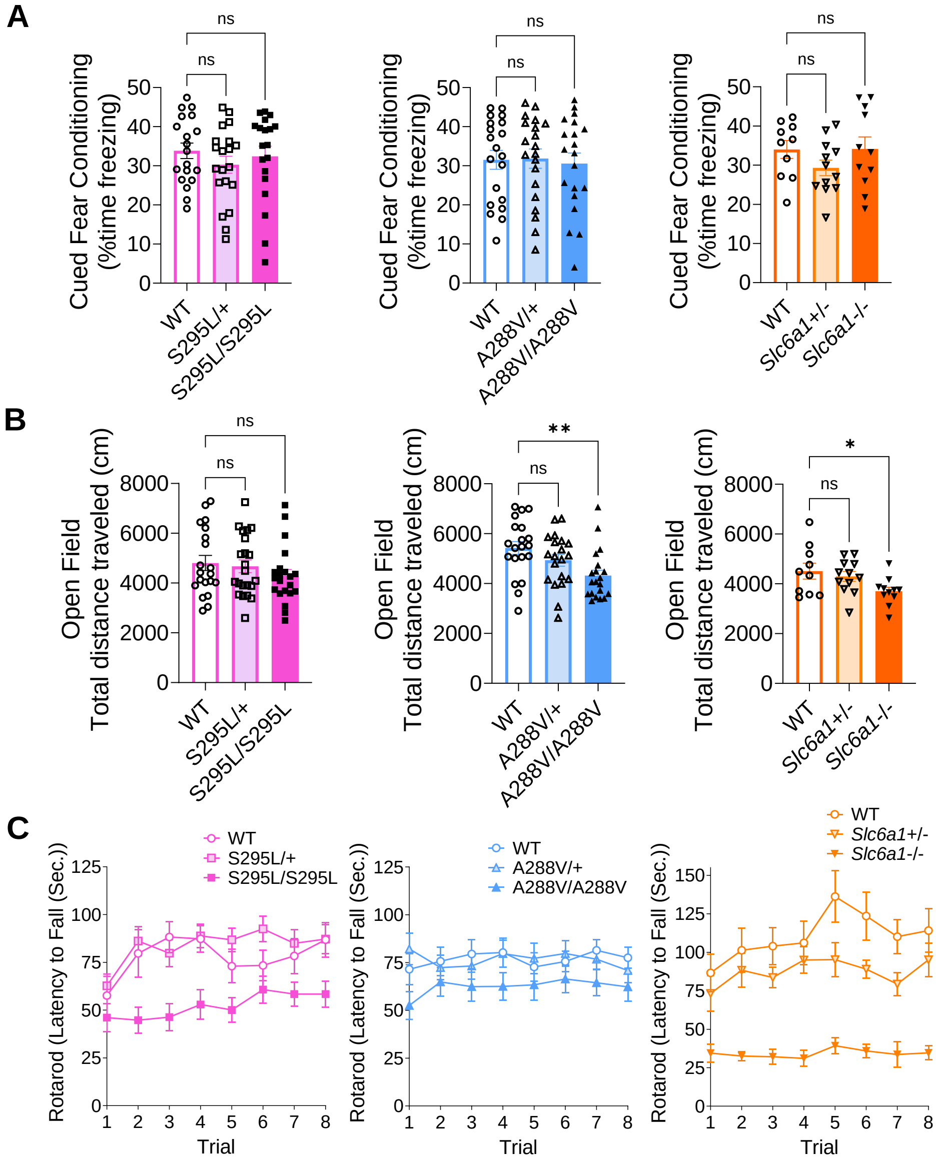

Supplemental Figure 2. **The cued fear conditioning, open field and rotarod tests in wildtype, heterozygous (*Slc6a1*$^{+/-}$, *Slc6a1*$^{S295L/+}$, *Slc6a1*$^{A288V/+}$) and homozygous (*Slc6a1*$^{-/-}$, *Slc6a1*$^{S295L/S295L}$, *Slc6a1*$^{A288V/A288V}$) *Slc6a1* KO and KI mice.** A) Freezing % time during the cued fear conditioning tests. B) Distance traveled in open field tests. C) Latency to fall (sec) across 8 trials of the rotarod tests. Sample number in A, B, and C, WT, *Slc6a1*$^{S295L/+}$, *Slc6a1*$^{S295L/S295L}$: n=19-20 per group; WT, *Slc6a1*$^{A288V/+}$, *Slc6a1*$^{A288V/A288V}$: n=20 per group; WT, *Slc6a1*$^{-/+}$, *Slc6a1*$^{-/-}$: n=10-12 per group. Data are means ± SEM, One-way ANOVA was used for statistical analysis, *P < 0.05, **P < 0.01.

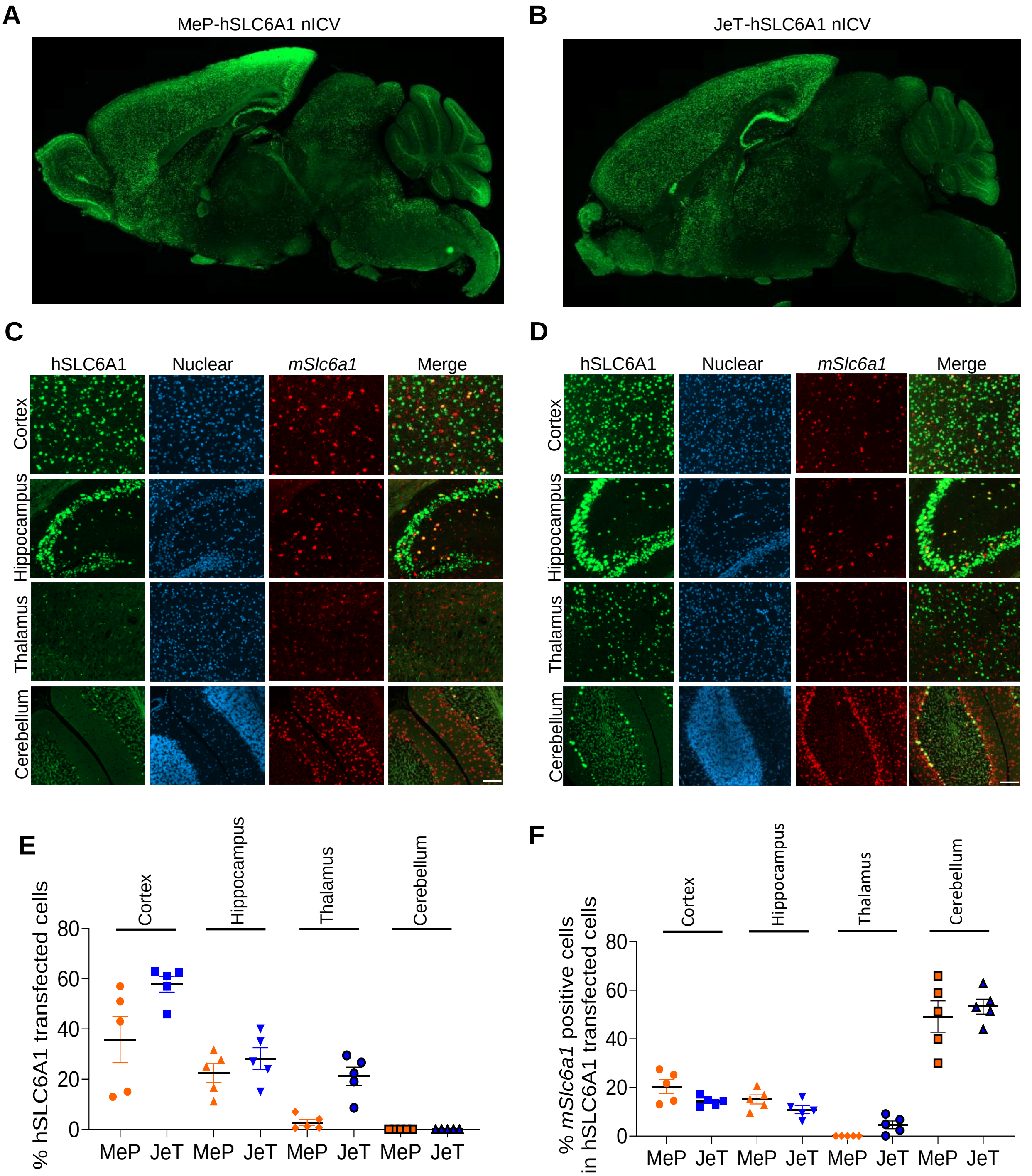

Supplemental Figure 3. **The mRNA expression pattern of the *hSLC6A1* transgene in the mouse brain via neonatal intracerebroventricular injection.** Neonatal *Slc6a1*$^{+/-}$ mice were administered either AAV9/MeP229-h*SLC6A1* or AAV9/JeT-*hSLC6A1* by the ICV route, then *hSLC6A1* transgene and/or endogenous murine *Slc6a1* expression across the brain was visualized by RNAscope at 4 to 6 months of age. A) RNAscope of *hSLC6A1* (green) following AAV9/MeP229-*hSLC6A1* treatment, counterstained with DAPI to label nuclei (blue). B) RNAscope of *hSLC6A1* (green) following AAV9/JeT-*hSLC6A1* treatment, counterstained with DAPI (blue). C) RNAscope of *hSLC6A1* (green) and murine endogenous *Slc6a1* (red) following AAV9/MeP229-*hSLC6A1* treatment, counterstained with DAPI (blue). Representative brain images are shown for the cortex, hippocampus, thalamus, and cerebellum (scale bar, 100um). D) RNAscope of *hSLC6A1* (green) and murine endogenous *Slc6a1* (red) following AAV9/JeT-*hSLC6A1* treatment, counterstained with DAPI (blue). Representative brain images are shown for the cortex, hippocampus, thalamus, and cerebellum (scale bar, 100um). E) Quantification of the percentage of human *SLC6A1* transduced cells in each brain region. The percentages of *hSLC6A1* transduced cells are expressed as a ratio of green positive cells to total counted nuclear DAPI positive cells number (counting at least 1000 nuclei of each sample/brain area); F) Quantification of the percentage of endogenous *mSlc6a1*-positive cells, in *hSLC6A1* transduced cells of each brain region (expressed as a ratio of the double SLC6A1 positive cells (red and green) to transduced SLC6A1 positive cells (green). Data are represented by the mean ± SEM, n=5 per group.

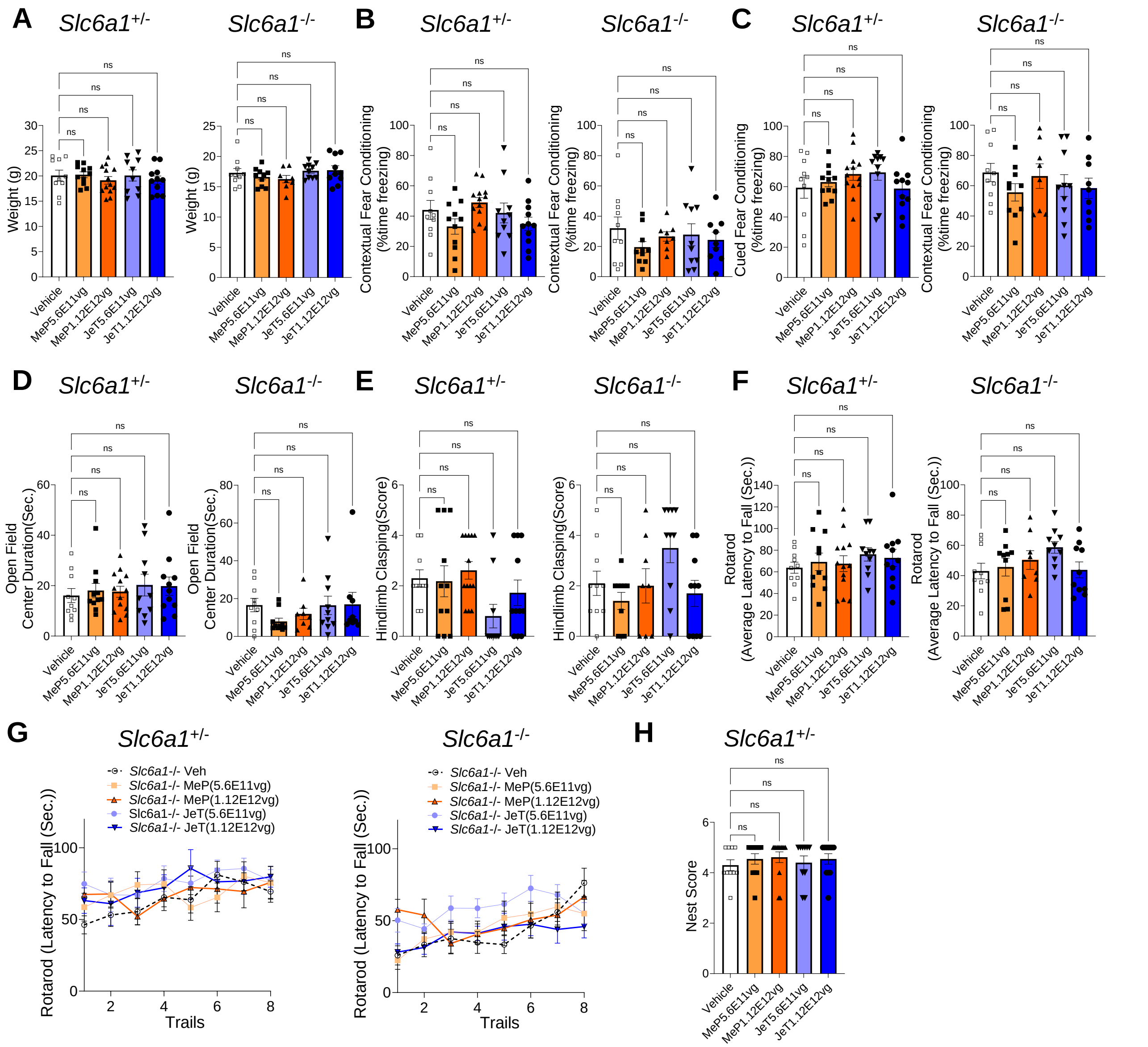

Supplemental Figure 4. Nest building, cued fear conditioning, body weights, open field, hindlimb clasping, and rotarod tests for *Slc6a1*$^{+/-}$ and *Slc6a1*$^{-/-}$ mice injected with vehicle, AAV9/MeP229-*hSLC6A1* or AAV9/JET-*hSLC6A1* at PND5 via IT injection. A)Body weights; B)Contextual fear conditioning freezing levels; C)Cued fear conditioning freezing levels ; D)Center time in an open field; E)Hindlimb clasping scores; F)Average rotarod latency to fall time and G)Latency to fall (sec) across 8 trials of the rotarod tests for treated *Slc6a1*$^{+/-}$ mice (left to right: n=10, 11, 13, 10 and 11) and *Slc6a1* $^{-/-}$ mice(left to right: n=10, 10, 8, 10 and 9). H) Nest building score in treated *Slc6a1*$^{+/-}$ mice(left to right: n=10, 11, 13, 10 and 11). Data are represented by the mean ± SEM. One-way ANOVA with Dunnett's multiple comparisons test was used for statistical analysis.

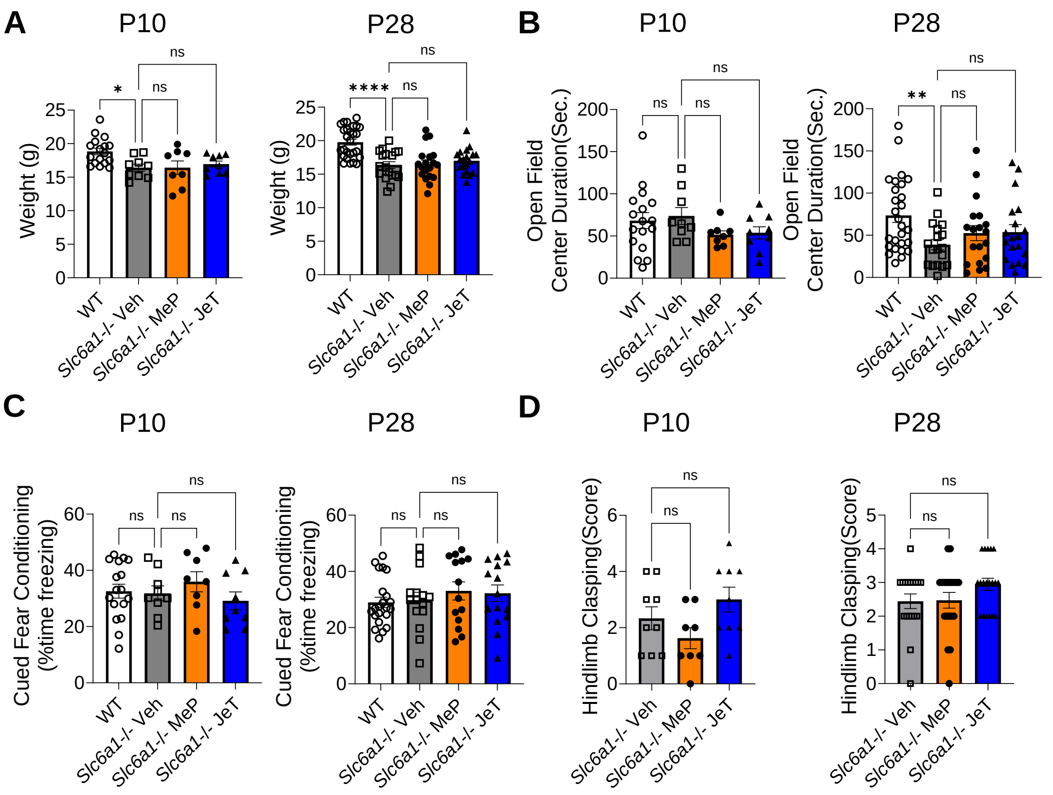

Supplemental Figure 5. Body weights, open field, cued fear conditioning, and hindlimb clasping tests for *Slc6a1*$^{-/-}$ KO mice injected IT with vehicle, AAV9/MeP229-*hSLC6A1* or AAV9/JeT-*hSLC6A1* at PND10 or PND28 as described in Figure 4. A) Body weights (left to right: n=17, 9, 9, 8, 27, 18, 19 and 19). B) Center time in an open field (left to right: n=17, 9, 9, 8, 27, 18, 19 and 19). C) Cued fear conditioning freezing levels (left to right: n=17, 9, 9, 8, 22, 15, 15 and 14). D) Hindlimb clasping scores (left to right: n=9, 9, 8, 18, 19 and 19).

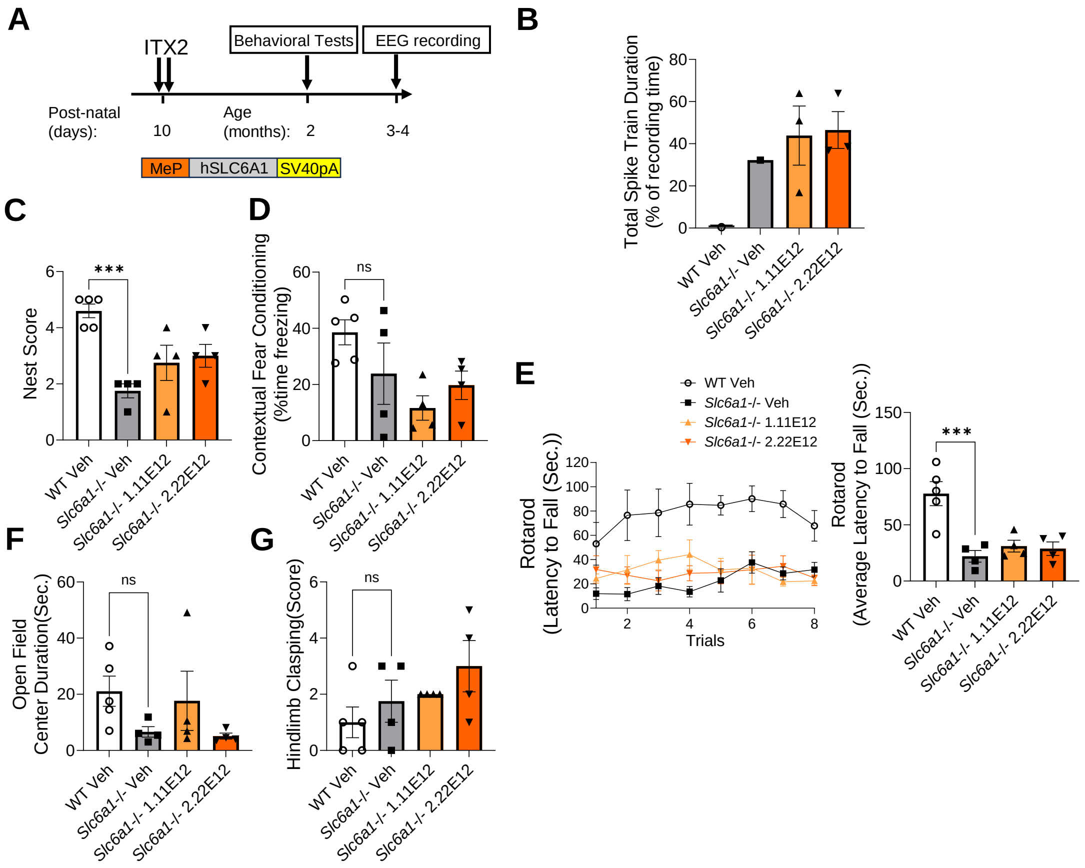

Supplemental Figure 6. Increased viral transduction via double IT administration to PND10 *Slc6a1*$^{-/-}$KO mice did not improve behavioral efficacy outcomes. A) Experimental design of preclinical AAV9/MeP229-*hSLC6A1* gene therapy via double IT injection to P10 ***Slc6a1***$^{-/-}$ KO mice (vehicle, 1.11x10^{12} vg, or 2.22x10^{12} vg). Study readouts at specified ages are listed. B) Quantification of total spike train duration across a 48 h EEG recording period (% recording time) for treated WT and ***Slc6a1***$^{-/-}$ mice (left to right: n=1, 1, 3 and 3). C) Nest building scores; D) Freezing % time during the contextual fear conditioning tests; E) Latency to fall (sec) across 8 trials and average latency to fall (sec, average of 8 trials) of the rotarod tests; F) Center time in an open field; G) Hindlimb clasping scores. Data are represented by the mean ± SEM, Sample number in C-G: n=4-5 each group. One-way ANOVA was used for statistical analysis.

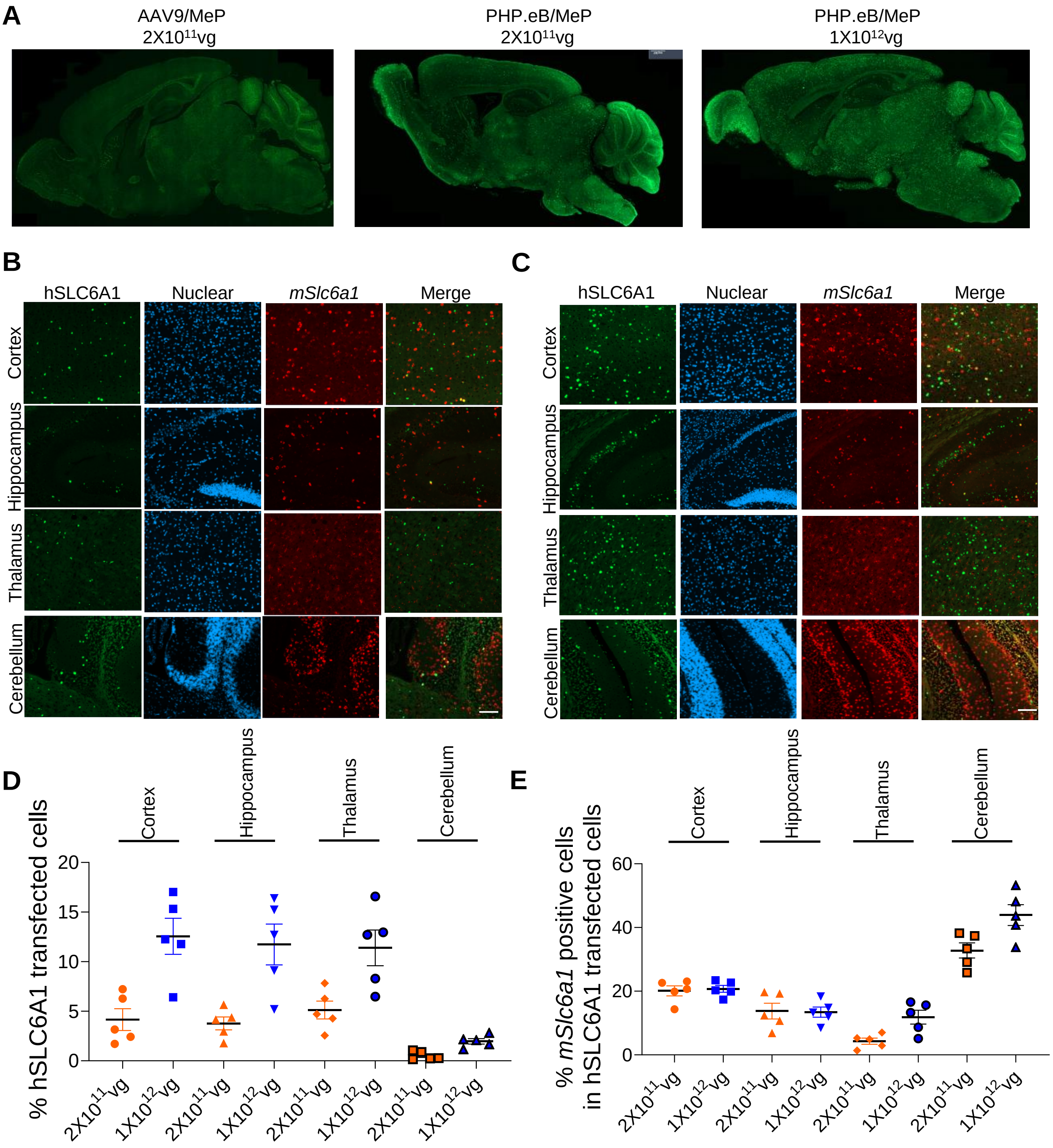

Supplemental Figure 7. The mRNA expression pattern of the *hSLC6A1* transgene in the brain after PHP.eB/MeP229-*hSLC6A1* IV injection in PND23 WT mice. A) RNAscope multiplex fluorescent images at **3-4 months** showing *hSLC6A1* mRNA expression (green) by PND23 IV injection with 2x10^{10}, 2x10^{11} or 1x10^{12} vg of PHP.eB/MeP229-*hSLC6A1*. B) RNAscope dual labelling of *hSLC6A1* transgene (green) and endogenous murine *Slc6a1*(red) mRNA from the 2x10^{10} vg dose, counterstained with DAPI to label nuclei (blue). Representative images are shown from cortex, hippocampus, thalamus, and cerebellum (scale bar, 100um). C) As with (B), representative images from the 2x10^{11} vg dose group (scale bar, 100um). D) Quantification of the percentage of transduced human *SLC6A1*-positive cells in *Slc6a1*$^{+/-}$ in the cortex. The percentage of *hSLC6A1* transduced cells are expressed as a ratio of, green *hSLC6A1*-positive cells, divided by the total number of counted blue DAPI-positive nuclei (counting at least 1000 nuclei of each sample/brain area); I) Quantification of the percentage of endogenous *mSlc6a1*-positive cells, in *hSLC6A1* transduced cells of each brain region (expressed as a ratio of the double SLC6A1 positive cells (red and green) to transduced SLC6A1 positive cells (green). Data are represented by the mean ± SEM, n=5 per group.

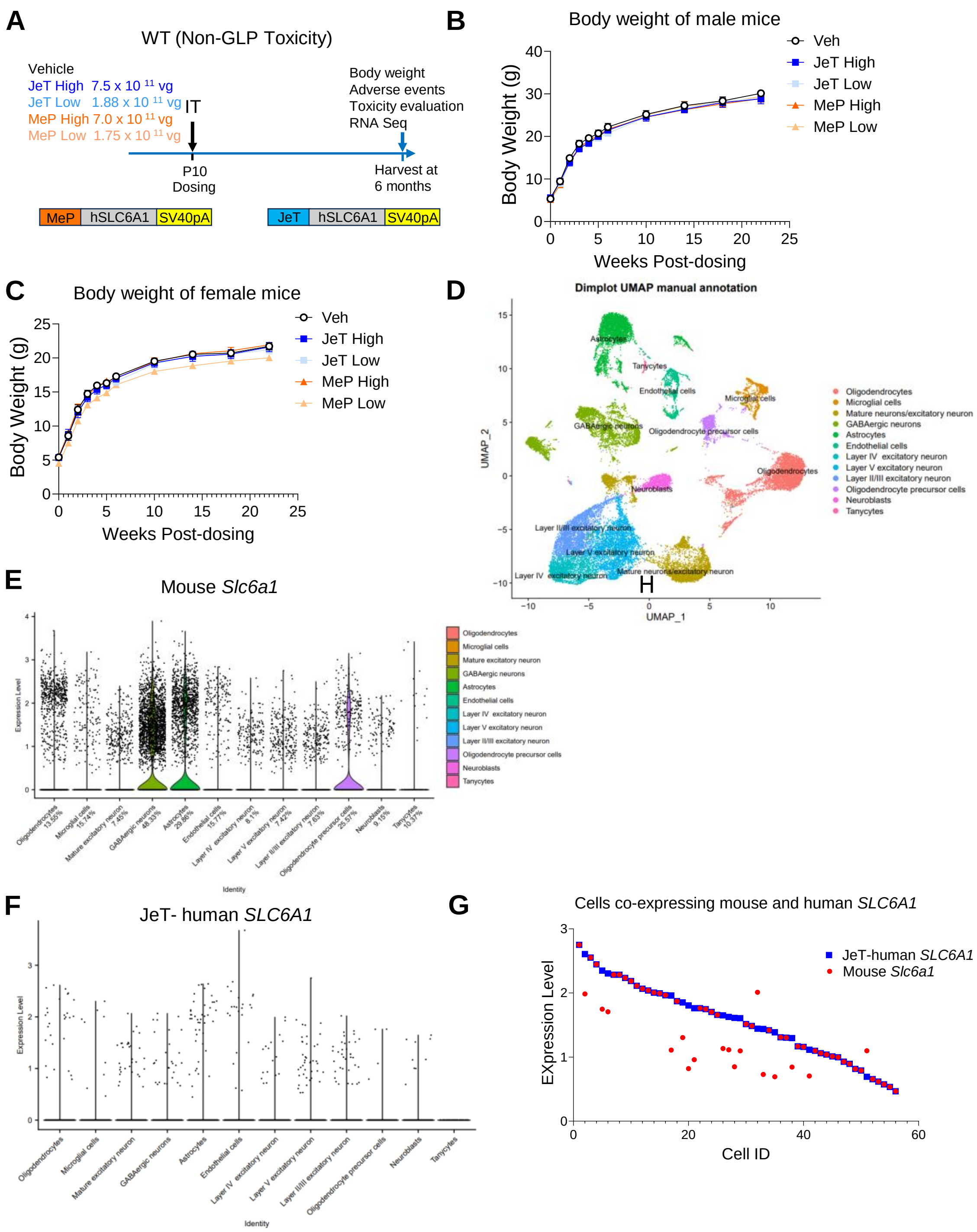

Supplemental Figure 8. IT administration of either AAV9/JeT-*hSLC6A1* or AAV9/MeP-*hSLC6A1* in WT mice at PND10 did not lead to any adverse effects up to 6 months post-injection. A) Experimental design of the non-GLP toxicology study in male and female WT C57Bl6/J mice injected with vehicle, AAV9/MeP229-*hSLC6A1* (1.75×10^{11} vg (Low) or 7.0×10^{11} vg (High)) and AAV9/JeT-*hSLC6A1* (1.88×10^{11} vg (Low) or 7.5×10^{11} vg (High)) via IT injection at PND28. Study readouts at each time point after dose administration or at specified age are listed. B) Longitudinal body weights of treated male mice, n=6 per group. C) Longitudinal body weights of treated female mice, n=6 per group. Single-nuclei RNA sequencing analysis conducted at 6 months old from a WT mouse brain cortex, following high dose (7.5×10^{11} vg) IT injection of AAV9/*JeT-hSLC6A1* vector at PND10. D) A UMAP plot is provided, displaying the results of unbiased clustering of 27375 single cell nuclei reads from brain cortex. E) Distribution of cells expressing endogenous murine *Slc6a1* mRNA (4979 positive cells) from different clusters in violin plot, percentage (the count expressed murine *Slc6a1* mRNA / the total count in cluster) is also added. F) Distribution of cells expressing *JeT-hSLC6A1* transgene mRNA (272 positive cells) from different clusters in violin plot. G) Relative mRNA expression level of 57 cells co-expressing human and mouse *Slc6a1* mRNA. All graphed data are shown as mean ± SEM. The count expressed gene/ count in cluster percentage is also added in violin plot.

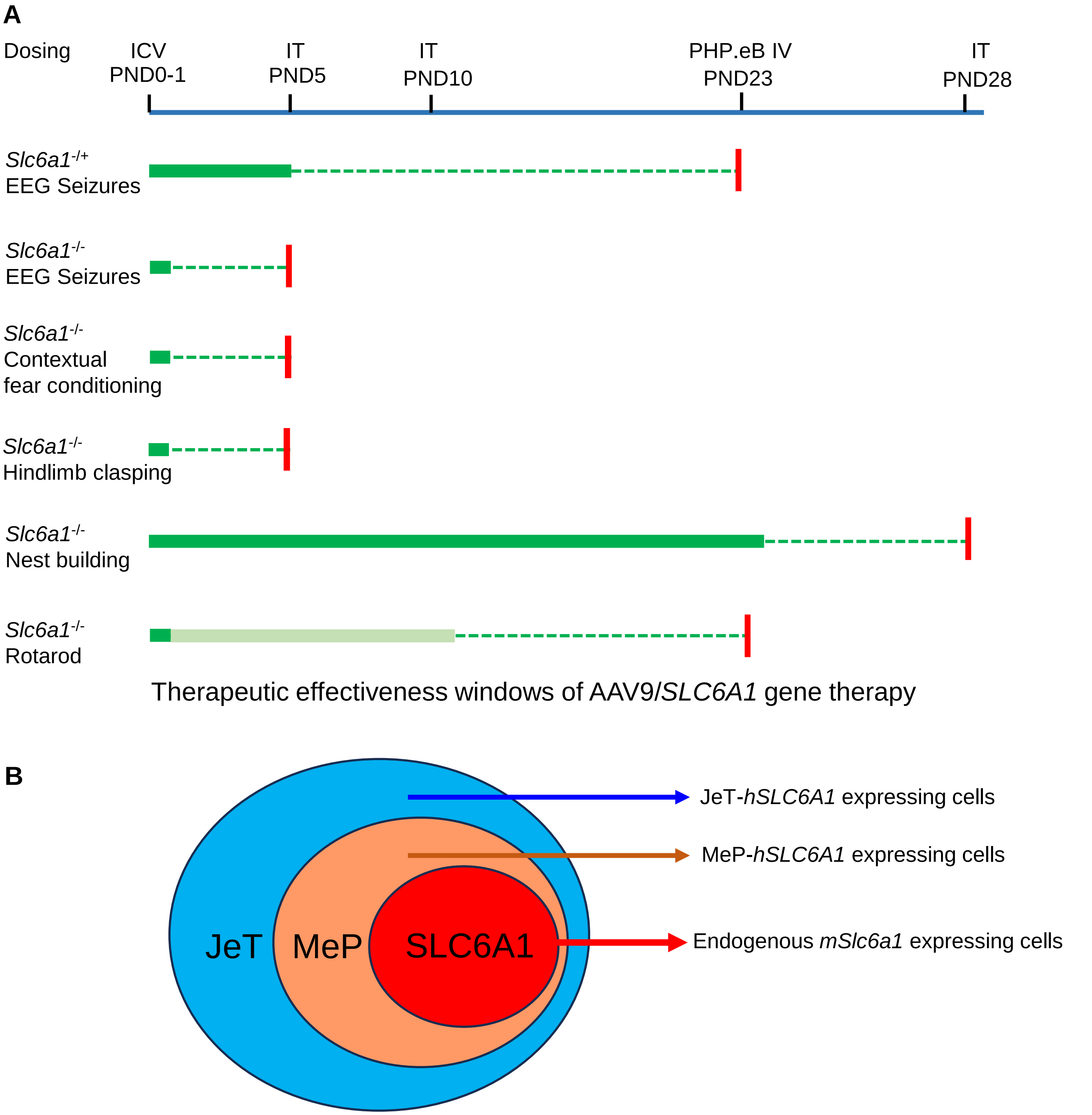

Supplemental Figure 9. Schematics models of AAV9/*SLC6A1* gene therapy discussion. A) Therapeutic effectiveness windows of AAV9/*SLC6A1* gene therapy in EEG seizures, contextual fear conditioning, nest building and rotarod phenotypes in heterozygous and homozygous *Slc6a1* KO mice(a solid green line indicates an improvement in *Slc6a1* mice phenotype; a light green line indicates a trend, but not significant for improvement in *Slc6a1* mice phenotype; a dashed green line indicates an unknown efficacy result; a red line indicates no improvement in *Slc6a1* mice phenotype). B) Schematics pattern of JeT promoter and MeP promoter expression cells in mouse brain.

Supplemental Table 1. *Slc6a1* mouse models mimic human *SLC6A1*-related disorders clinical symptoms

Phenotype severity	Heterozygous mouse models: Mild			Intermediate	Homozygous mouse models: Severe		
	**A288V HET**	**S295L HET**	***Slc6a1* HET**	**Human Clinical Phenotyping**	**A288V KI**	**S295L KI**	***Slc6a1* KO**
	Slc6a1$^{A288V/+}$<br>(Partial LOF)<br>~60.75% SLC6A1 function(*23*)	*Slc6a1*$^{S295L/+}$<br>(LOF)<br>~50% SLC6A1 function(*11*)	*Slc6a1*$^{+/-}$<br>~50% SLC6A1 function	SLC6A1 haploinsufficiency<br>~50% SLC6A1 function	*Slc6a1*$^{A288V/A288V}$<br>(Partial LOF)<br>~20% SLC6A1 function	*Slc6a1*$^{S295L/S295L}$<br>(LOF)<br>~0% SLC6A1 function	*Slc6a1*$^{+/-}$<br>0% SLC6A1 function
	EEG abnormality	EEG abnormality	EEG abnormality	Epilepsy<br>(EEG abnormality)	Severe EEG abnormality	Severe EEG abnormality	Severe EEG abnormality
	Normal Body weight	Normal Body weight	Normal Body weight	Developmental delay	Reduced Body weight	Reduced Body weight	Reduced Body weight
	Normal contextual fear conditioning<br>Normal nest building,<br>Normal open field	Normal contextual fear conditioning<br>Normal nest building,<br>Normal open field	Normal contextual fear conditioning,<br>Normal nest building,<br>Normal open field	Cognitive impairment,<br>Behavioral disorders,<br>Autistic traits	Normal contextual fear conditioning(Trend, n.s.),<br>Poor nest building,<br>Anxiety in Open Field test	Reduced contextual fear conditioning,<br>Poor nest building,<br>Anxiety in Open Field test	Reduced contextual fear conditioning,<br>Poor nest building,<br>Anxiety in Open Field test
	Normal Rotarod performance,<br>No Hindlimb clasping phenotype	Normal Rotarod performance,<br>No Hindlimb clasping phenotype	Normal Rotarod performance,<br>No Hindlimb clasping phenotype	Motor dysfunction	Normal Rotarod performance,<br>No Hindlimb clasping phenotype	Decreased Rotarod performance,<br>Hindlimb clasping phenotype	Decreased Rotarod performance,<br>Hindlimb clasping phenotype

Supplemental Table 2. Adverse effects of neonatal ICV AAV9/ JeT-*hSLC6A1* and MEP-*hSLC6A1* injections in *Slc6a1* HET and KO mice

Neonatal ICV Injection (mice number)	Vehicle(n= 46) 23 HET, 23 KO	AAV9/MeP-SLC6A1(n= 45) 13 HET, 29 KO, 3 unknown	AAV9/JET-SLC6A1 (n= 19) 11 HET, 6 KO, 2 unknown
Death	**1(2.1%)** (1-KO)	**8 (17.7%)** (2-KO, 3-HET, 3-Pre-weaning (not genotyped))	**9(47.3%)** (3-KO, 3-HET, 3-Pre-weaning(2 not genotyped))
Seizures (Convulsive seizures)	**0**	**6(13.3%)** (4-KO, 2-HET)	**4(21%)** (2-KO, 2-HET)

Supplemental Table 3. Summary of adverse events at different developmental ages of WT, *Slc6a1*$^{+/-}$ and *Slc6a1*$^{-/-}$ mice treated with Vehicle, MeP-*hSLC6A1* and JeT-*hSLC6A1* vector.

WT mice

Viral vector	Safety adverse event	Mouse age (days) of gene therapy treatment											
		P0-1	P5		P10				P23(PHP.eB)			P28	
		3E11	5.6E11	1.1E11	1.8E11	7.5E11	1.1E11	2.2E11	2E10	2E11	1E12	1.8E11	7.5E11
JeT-SLC6A1	Death	NT	NT	NT	**0/6**	**0/6**	NT	NT	**0/6**	**0/6**	**0/6**	1/12	0/12
	Adverse event	NT	NT	NT	**0/6**	**0/6**	NT	NT	**0/6**	**0/6**	**0/6**	0/12	0/12
MeP-SLC6A1	Death	NT	NT	NT	**0/6**	**0/6**	NT	NT	**0/6**	**0/6**	**0/6**	0/12	0/12
	Adverse event	NT	NT	NT	**0/6**	**0/6**	NT	NT	**0/6**	**0/6**	**0/6**	0/12	0/12
Vehicle	Death	NT			0/11				0/31			0/12	
	Adverse event	NT			0/11				0/31			0/12	

Slc6a1$^{+/-}$ and *Slc6a1*$^{-/-}$ mice

Viral vector	Safety adverse event	Mouse age (days) of gene therapy treatment											
		P0-1	P5		P10				P23(PHP.eB)			P28	
		3E11	5.6E11	1.1E11	1.8E11	7.5E11	1.1E11	2.2E11	2E10	2E11	1E12	1.8E11	7.5E11
JeT-SLC6A1	Death	9/19	1/22	1/23	NT	0/9	NT	NT	**NT**	**NT**	**NT**	NT	1/20
	Adverse event	4/19[a]	1/22[c]	1/23[d]	NT	0/9	NT	NT	NT	NT	NT	NT	0/20
MeP-SLC6A1	Death	8/45	0/22	0/21	NT	0/8	0/4	0/4	0/32	0/33	0/10	NT	0/19
	Adverse event	6/45[a]	0/22	0/21	NT	0/8	0/4	0/4	0/32	0/33	0/10	NT	0/19
Vehicle	Death	1/46	0/20		0/13		0/4		0/29			0/18	
	Adverse event	0/46	0/20		0/13		0/4		0/29			0/18	

Adverse event: a: convulsive seizures; c, non-intervention related hydrocephalus; d, very small, thin, hunched; NT, not tested.

Supplemental Table 4. Summary of MeP-*SLC6A1* and JeT-*SLC6A1* efficacy study at different developmental ages of *Slc6a1*$^{+/-}$ and *Slc6a1*$^{-/-}$ mice.

Slc6a1$^{+/-}$ heterozygous mouse mimics human *SLC6A1* haploinsufficiency disorders clinical symptoms in abnormal EEG seizures.

SLC6A1-related disorders clinical symptoms	SLC6A1$^{+/-}$ HET mouse	Viral vector	Mouse age(days) of gene therapy treatment				
			**P0-1**	**P5**	**P10**	**P21(PHP.eB)**	**P28**
EEG abnormality	EEG abnormality	MeP	Full rescue	Full rescue	NT	**No rescue**	NT
		JeT	Full rescue	Full rescue	NT	NT	NT
Developmental delay	Not observed	N/A					
Cognitive impairment, Behavioral disorders, Autistic traits	No behavioral phenotypes						
Motor dysfunction	No behavioral phenotypes						

Homozygous *Slc6a1*$^{-/-}$ KO mouse displays severe EEG seizures and human *SLC6A1* haploinsufficiency disorders-like abnormal behaviors

SLC6A1-related disorders clinical symptoms	SLC6A1$^{-/-}$ KO mouse	Viral vector Promoter	Mouse age(days) of gene therapy treatment				
			**P0-1**	**P5**	**P10**	**P21(PHP.eB)**	**P28**
EEG abnormality	Severe EEG abnormality	MeP	Partial rescue	**No rescue**	**No rescue**	**No rescue**	**No rescue**
		JeT	Full rescue	**No rescue**	**No rescue**	NT	**No rescue**
Developmental delay	Reduced Body weight	MeP	**No rescue**	**No rescue**	**No rescue**	**No rescue**	**No rescue**
		JeT	**No rescue**	**No rescue**	**No rescue**	NT	**No rescue**
Cognitive impairment, Behavioral disorders, Autistic traits	Reduced contextual fear conditioning	MeP	Rescue	**No rescue**	**No rescue**	**No rescue**	**No rescue**
		JeT	NT	**No rescue**	**No rescue**	NT	**No rescue**
	Poor nest building	MeP	Rescue	Rescue	Improved(n.s.)	Rescue	**No rescue**
		JeT	NT	Rescue	Rescue	NT	**No rescue**
	Anxiety in Open Field test	MeP	Improved(n.s.)	**No rescue**	**No rescue**	**No rescue**	**No rescue**
		JeT	NT	**No rescue**	**No rescue**	NT	**No rescue**
Motor dysfunction	Decreased Rotarod performance	MeP	Rescue	**No rescue**	Improved(n.s.)	**No rescue**	**No rescue**
		JeT	NT	Improved(n.s.)	Improved(n.s.)	NT	**No rescue**
	Hindlimb clasping	MeP	Rescue	**No rescue**	**No rescue**	**No rescue**	**No rescue**
		JeT	NT	**No rescue**	**No rescue**	NT	**No rescue**

NT, not tested; n.s. not significant
